# Hypoxia sensing requires H_2_S-dependent persulfidation of olfactory receptor 78

**DOI:** 10.1126/sciadv.adf3026

**Published:** 2023-07-05

**Authors:** Ying-Jie Peng, Jayasri Nanduri, Ning Wang, Ganesh K. Kumar, Vytautas Bindokas, Bindu D. Paul, Xuanmao Chen, Aaron P. Fox, Thibaut Vignane, Milos R. Filipovic, Nanduri R. Prabhakar

**Affiliations:** ^1^ Institute for Integrative Physiology, Biological Sciences Division, University of Chicago, Chicago, IL, USA.; ^2^ Department of Physiology and Pharmacological Sciences, Biological Sciences Division, University of Chicago, Chicago, IL, USA.; ^3^ Department of Pharmacology, The Johns Hopkins University, Baltimore, MD, USA.; ^4^ Department of Molecular, Cellular and Biomedical Sciences, College of Life Sciences and Agriculture, University of New Hampshire, Durham, NH USA.; ^5^ Leibniz-Institut für Analytische Wissenschaften–ISAS, Bunsen-Kirchhoff-Straße, 1144139 Dortmund, Germany.

## Abstract

Oxygen (O_2_) sensing by the carotid body is critical for maintaining cardiorespiratory homeostasis during hypoxia. Hydrogen sulfide (H_2_S) signaling is implicated in carotid body activation by low O_2_. Here, we show that persulfidation of olfactory receptor 78 (Olfr78) by H_2_S is an integral component of carotid body activation by hypoxia. Hypoxia and H_2_S increased persulfidation in carotid body glomus cells and persulfidated cysteine^240^ in Olfr78 protein in heterologous system. *Olfr78* mutants manifest impaired carotid body sensory nerve, glomus cell, and breathing responses to H_2_S and hypoxia. Glomus cells are positive for G_Olf,_ adenylate cyclase 3 (Adcy3) and cyclic nucleotide–gated channel alpha 2 (Cnga2), key molecules of odorant receptor signaling. *Adcy3* or *Cnga2* mutants exhibited impaired carotid body and glomus cell responses to H_2_S and breathing responses to hypoxia. These results suggest that H_2_S through redox modification of Olfr78 participates in carotid body activation by hypoxia to regulate breathing.

## Teaser

Hypoxia sensing by the carotid body involves hydrogen-sulfide dependent oxidative modification of Olfr78 protein.

## INTRODUCTION

Carotid bodies are sensory organs for monitoring arterial blood O_2_ concentrations. Hypoxemia (reduced arterial blood O_2_ concentrations) stimulates neural activity of the carotid body, triggering reflex stimulation of breathing and blood pressure, events critical for maintaining homeostasis under low O_2_ environment. The sensory unit of the carotid body comprises O_2_-sensitive glomus cells and the nearby sensory nerve ending (*1*). Signaling mechanism(s) transducing blood O_2_ levels to increased carotid body neural activity are not well-understood (*1*–*3*).

Glomus cells are positive for cystathionine-γ-lyase (CSE), a hydrogen sulfide (H_2_S)–synthesizing enzyme (*4*, *5*). Hypoxia increases H_2_S abundance in the carotid body in a stimulus-dependent manner (*4*). Mice with global deletion of the *Cth* gene encoding CSE exhibit absence of increased H_2_S abundance by hypoxia and impaired carotid body neural activation, glomus cell responses, and stimulation of breathing by low O_2_ (*4*–*6*). These studies suggest H_2_S as an important mediator of carotid body activation by hypoxia. However, signaling mechanism(s) underlying H_2_S-dependent activation of the carotid body by hypoxia is not known.

Murine glomus cells express high abundance of the gene encoding olfactory receptor 78 (*Olfr78*) (*7*, *8*). Chang *et al.* (*7*) reported impaired carotid body sensory nerve (CSN), glomus cell, and breathing response to hypoxia in *Olfr78*-null mice. It was proposed that CSN activation by hypoxia requires Olfr78 activation by lactate. However, Torres-Torrelo *et al.* (*9*) reported that Olfr78-null mice manifest unaltered breathing responses, as well as [Ca^2+^]_i_ and transmitter secretion from glomus cells in response to hypoxia (P_O___2__ ~ 10 to 15 mmHg) and lactate. These findings questioned the role of Olfr78-lactate signaling in carotid body activation by hypoxia. Reassessment of CSN, glomus [Ca^2+^]_i_, and breathing response of *Olfr78* mutants showed that all these responses are impaired in response to a wide range of hypoxia but not to severe hypoxia (P_O_2__ ~10 mmHg) (*10*), or CO_2_ (*11*). These findings suggest participation of Olfr78 in hypoxic but not severe hypoxia sensing by the carotid body. Although lactate and short-chain fatty acids (SFAs) are ligands for Olfr78 (*7*, *12*), CSN and glomus responses to either lactate or SFAs were unaltered in *Olfr78*^−/−^ mutants (*10*). Thus, the mechanisms underlying Olfr78 activation by hypoxia remain elusive.

H_2_S is a gaseous molecule with a distinct odor. Olfr78 belongs to an odorant receptor family. Current study tested the hypothesis that H_2_S activates Olfr78, and the ensuing G protein–coupled receptor (GPCR) signaling governs carotid body hypoxic sensing.

## RESULTS

### H_2_S activates Olfr78

**Fig. 1.**
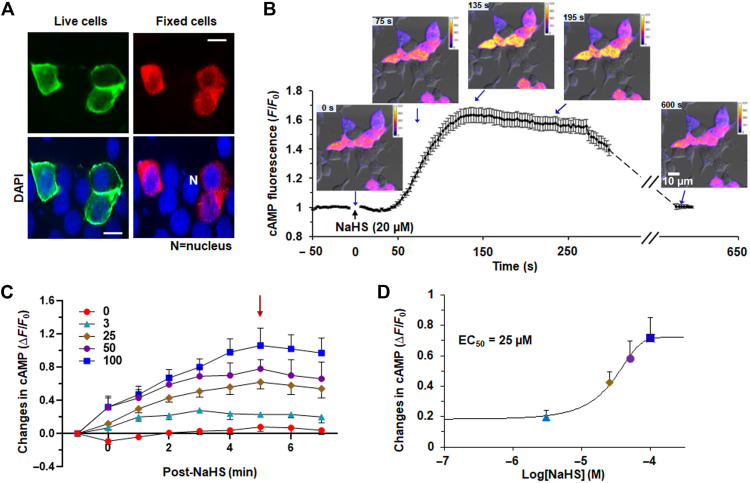
NaHS activates Olfr78. (**A**) Detection of Olfr78 at the cell surface of live cells (top left) and at the cytosol of fixed cells (top right) in HEK293 cells transfected with flag-Olfr78 plasmid. Bottom: Nuclear staining with 4′,6-diamidino-2-phenylindole (DAPI) of the same cells. Scale bars, 10 μm. (**B**) Temporal response of cAMP fluorescence in HEK293 cells transfected with Olfr78 plasmid in response to NaHS. cAMP fluorescence was monitored with a microscope. Quantitative data (means ± SEM from 25 cells) and examples of images of cAMP fluorescence changes in the intensity of cAMP fluorescence in HEK293 cells before and after application of NaHS (20 μM) are shown. (**C**) Olfr78 activation by the indicated concentrations of NaHS (in micromolar) measured as changes in the intensity of cAMP fluorescence and presented as the ratio of absolute change (stimuli-baseline) over baseline (Δ*F*/*F*_0_). The arrow indicates the peak response of cAMP fluorescence. Significant differences were calculated by two-way analysis of variance (ANOVA) with repeated measures followed by Holm-Sidak’s test. Changes in cAMP fluorescence were significantly different with time after NaHS application [*F*(8189) = 13.082, *P* < 0.001] and concentration of NaHS [*F*(4189) = 146.224, *P* < 0.001]. Data represent means ± SEM; *n* = 5 to 6 measurements for each concentration of NaHS. (**D**) Concentration-response curve of changes in cAMP fluorescence integral of the total response for 0 to 5 min. Data represent means ± SEM; *n* = 5 to 6 individual measurements with each concentration.

We first determined the effect of H_2_S on Olfr78 using sodium hydrogen sulfide (NaHS), a salt form of H_2_S. Human embryonic kidney (HEK)–293 cells were transfected with a flag-tagged Olfr78 plasmid. Transfection efficiency ranged between ~25 and 30%. Flag-Olfr78 was abundant at the cell surface on the basis of live cell immunofluorescence (Fig. 1A), indicating that Olfr78 trafficked to the plasma membrane even in the absence of receptor-specific chaperone proteins. In fixed cells, flag-Olfr78 was detected in the cytosol as well (Fig. 1A). Although a cyclic adenosine monophosphate (cAMP)–driven cAMP response element–binding protein (CREB) luciferase assay was used for assessing Olfr78 activation by lactate or SFAs (*12*, *13*), the long incubation required for this assay makes it unsuitable for evaluating effects of H_2_S, which has a half-life ranging from seconds to minutes (*14*, *15*). Therefore, we used a fluorescent biosensor to measure cAMP responses to NaHS (*16*). We detected a rapid (within seconds) increase in cAMP fluorescence in response to 20 μM NaHS in cells expressing Olfr78, and this fluorescence lasted for about ~5 min and returned to baseline after ~10 min (Fig. 1B). Increasing concentrations of NaHS produced a progressive increase in cAMP fluorescence with peak response at 5 min after the application (Fig. 1C) and a median effective concentration (EC_50_) of 25 μM (Fig. 1D). We detected an NaHS-stimulated increase in cAMP fluorescence in cells expressing OR51E2, a human ortholog of mouse Olfr78, but not in cells expressing a mouse odorant receptor that is unrelated to Olfr78 (fig. S1A). cAMP fluorescence increased in response to isoproterenol, an activator of G_s_-coupled adrenergic receptors, and sodium acetate, an established Olfr78 ligand (fig. S1B) (*12*).

### H_2_S and hypoxia increase persulfide labeling in the carotid body

**Fig. 2.**
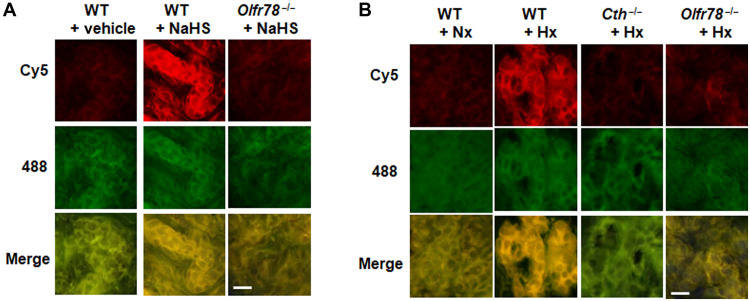
NaHS and hypoxia persulfidates Olfr78 in carotid bodies. (**A** and **B**) Example of microscopy images of persulfidation in glomus cells of the carotid body sections in response to 50 μM NaHS (A) or hypoxia (B), taken from wild-type (WT) or *Olfr78^−/−^* or *Cth ^−/−^* mice. The Cy5 signal corresponds to protein persulfides and 488-nm signal to NBF adducts. Scale bars, 10 μm. Carotid bodies were treated with either 50 μM NaHS or hypoxia (Hx; P_O___2__ ~ 40 mmHg) for 5 min. *n* = 3 experiments with each genotype.

Persulfidation (also called S-sulfhydration) of cysteine residues in target proteins is a major mechanism for biological actions of H_2_S (*17*, *18*). To demonstrate persulfidation of Olfr78 by H_2_S and hypoxia in glomus cells, the major O_2_ sensing cells of the carotid body, methanol-fixed carotid body sections were stained for persulfide labeling using the dimedone switch method as described (*18*). NaHS (50 μM) increased persulfidation as indicated by increased Cy5 signal in wild-type (WT) but not in *Olfr78* mutant glomus cells (Fig. 2A). Hypoxia (P_O___2__ ~ 40 mmHg), which increases H_2_S abundance in the carotid body (*4*) also increased persulfide signal in WT glomus cells, and this effect was absent on carotid bodies of *Cth* (encoding CSE; a major H_2_S synthesizing enzyme in the carotid body) and *Olfr78* mutants (Fig. 2B).

### NaHS persulfidates Cys^240^ of Olfr78

**Fig. 3.**
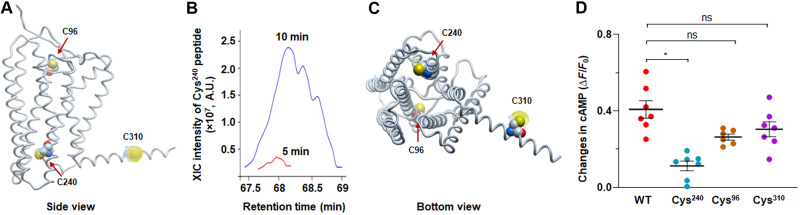
NaHS persulfidates Cys^240^ in Olfr78. (**A**) Side view of alpha fold–predicted Olfr78 structure. Cys^96^, Cys^240^, and Cys^310^ are highlighted using Corey-Pauling-Koltum (CPK) presentation with dot surface availability. (**B**) Extracted ion chromatograms (XIC) intensities for Cys^240^ peptides originating from Olfr78 isolated from the cells treated with NaHS (25 μM) for 5 or 10 min, indicating Cys^240^ as a major target of persulfidation by H_2_S. (**C**) Alpha fold–predicted Cys^240^ location in the interface of the cytosol and the membrane. (**D**) cAMP response to 25 μM NaHS in HEK293 cells expressing Olfr78 mutant with substitution of Cys^240^, Cys^96^, and Cys^310^ with alanine. Average (means ± SEM) and individual data from *n* = 6 to 7 individual experiments with WT and mutant cells. *P < 0.05; not significant (ns), *P* > 0.05; one-way ANOVA on ranks followed by Dunn test.

Primary sequence of mouse Olfr78 protein revealed nine cysteine residues (fig. S2). Persulfidation labeling by histochemistry is inadequate for identifying Cys residues affected by NaHS. To identify the cysteine residues affected by H_2_S, HEK293 cells transfected with a flag-tagged Olfr78 plasmid were treated with 25 μM NaHS for either 5 or 10 min. Cells were lysed with iodoacetamide (IAM) to block thiols and persulfides, and Olfr78 was immuno-pulled with anti-flag antibody immobilized by magnetic beads and digested by trypsin and/or chymotrypsin. The alpha fold model suggested Cys^96^, Cys^240^, and Cys^310^ in the core of Olfr78 protein (Fig. 3A). Tandem mass spectrometry (MS/MS) analysis showed increased persulfidation of Cys^240^ with NaHS in a time-dependent manner (Fig. 3B and fig. S3). Inhibitors of endogenous H_2_S production markedly reduced Cys^240^ persulfidation in Olfr78 expressing HEK cells (fig. S4). The alpha fold model suggested Cys^240^ in the interface of the cytosol and the membrane of Olfr78 (Fig. 3C). Substituting Cys^240^ with alanine blocked Olfr78 activation by NaHS as indicated by absence of increased cAMP fluorescence in HEK293 cells (Fig. 3D) and impaired Olfr78 protein trafficking to the cell membrane (fig. S5), consistent with the alpha fold model prediction of Cys^240^ location near the surface of the Olfr78. However, substituting Cys^96^ and Cys^310^ with alanine had no effect on cAMP fluorescence by NaHS (Fig. 3D). Collectively, these data suggested that Cys^240^ of the Olfr78 is a target of persulfidation by NaHS.

### Carotid body response to H_2_S does not involve mitochondria

H_2_S can affect biological functions also by inhibiting the mitochondrial electron transport chain (ETC) and ensuing elevation of mitochondrial reactive oxygen species (ROS) (*19*). Recent studies implicated mitochondrial ETC and mitochondrial ROS in glomus cell response to hypoxia (*20*, *21*). To assess the role of mitochondrial ROS generated by NaHS, CSN responses to NaHS (50 μM) and hypoxia (P_O___2__ ~ 40 mmHg) were determined before and 20 to 30 min after treating carotid bodies with Mito-TEMPO (20 μM), a scavenger of mitochondrial ROS. CSN activations either by NaHS or hypoxia were unaltered by Mito-TEMPO compared to vehicle-treated controls (fig. S6, A to D).

We next monitored mitochondrial membrane potential (MMP) as an index of mitochondrial ETC function with rhodamine-123 in mouse glomus cells as described (*22*). As positive controls, MMP response to 5 μM 2-{2-[4-(trifluoromethoxy) phenyl]hydrazinylidene}-propanedinitrile (FCCP) and hyperpolarization by oligomycin complex (20 μg/ml), a blocker of mitochondrial adenosine triphosphate synthetase, were recorded (fig. S7A). The 1 and 50 μM NaHS had no effect on MMP, whereas 300 μM NaHS depolarized MMP by ~60% relative to FCCP (fig. S7, B and C). A 50 μM NaHS, which had no effect on MMP, increased CSN activity, whereas 300 μM NaHS that produced robust depolarization of MMP caused only a brief and small excitation followed by inhibition of CSN activity (fig. S7D). The findings suggest inconsistency between CSN and MMP responses to NaHS.

### H_2_S-Olfr78 interaction governs carotid body hypoxic sensing

**Fig. 4.**
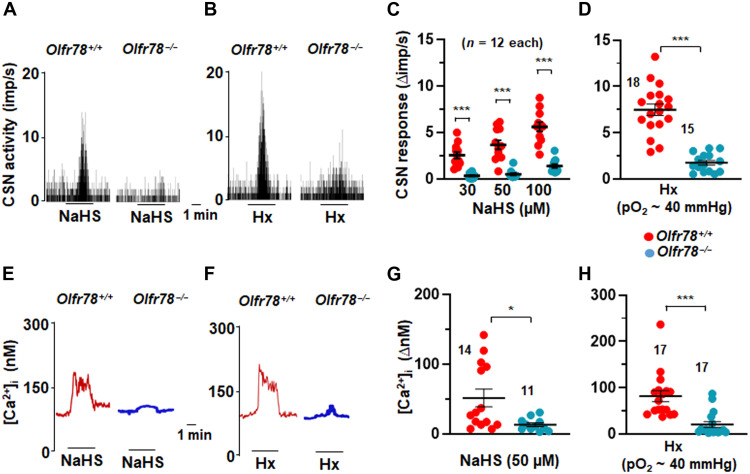
NaHS and hypoxia activate carotid body and glomus cells. (**A** and **B**) Examples of CSN responses to 50 μM NaHS (A) and hypoxia (Hx; P_O___2__ ~ 40 mmHg) (B) in *Olfr78*^+/+^ and *Olfr78^−/−^* mice. (**C**) Average (means ± SEM) and individual data of CSN responses to increasing concentrations of NaHS presented as the change in impulses per second (Δimp/s) in *Olfr78^+/+^* and *Olfr78^−/−^* mice. (**D**) Average (means ± SEM) and individual data of CSN responses to hypoxia presented as Hx minus baseline sensory nerve activity (Δimp/s) in *Olfr78^+/+^* and *Olfr78^−/−^* mice. (**E** to **H**) Example [Ca^2+^]_i_ responses of glomus cells to NaHS (50 μM) (E) and hypoxia (P_O___2__ ~ 40 mmHg) (F) in *Olfr78^+/+^* and *Olfr78^−/−^* mice. Average (means ± SEM) and individual data of [Ca^2+^]_i_ responses to NaHS in (G) and hypoxia in (H) in *Olfr78^+/+^* and *Olfr78^−/−^* mice. *P < 0.05 and ***P < 0.001; two-way ANOVA with repeated measures followed by Holm-Sidak test in (C) and Mann-Whitney test in (D), (G), and (H). In (C) and (D), *n* represents number of carotid bodies, and in (G) and (H), numbers represent the number of cells.

The importance of Olfr78 in CSN and glomus cell responses to NaHS or hypoxia was determined. Changes in CSN activity of the ex vivo carotid bodies and intracellular calcium concentration ([Ca^2+^]_i_) in the isolated glomus cells were determined in WT and *Olfr78*-null mice. Desired concentrations of NaHS were added to the fluid reservoirs irrigating the carotid body or glomus cells. We first determined the optimal concentration of NaHS affecting CSN activity. The 1 and 10 μM NaHS had no effect, and 30 μM NaHS stimulated CSN activity (fig. S8, A and B). CSN activity of WT mice increased in response to 30, 50, and 100 μM NaHS in a dose-dependent manner, and by hypoxia (P_O___2__ ~ 40 mmHg) but not in *Olfr78* mutants (Fig. 4, A to D). On the basis of these data, we chose 50 μM NaHS in further experiments.

At the cellular level, NaHS or hypoxia increased [Ca^2+^]_i_ in glomus cells from WT but not from *Olfr78*-null mice (Fig. 4, E to H). However, CSN stimulation by either CO_2_ or by sodium cyanide (NaCN), a pharmacological activator of the carotid body, was similar in carotid bodies from *Olfr78*-null or WT mice (fig. S9, A to D). These results demonstrated that carotid body responses to NaHS and hypoxia require Olfr78.

**Fig. 5.**
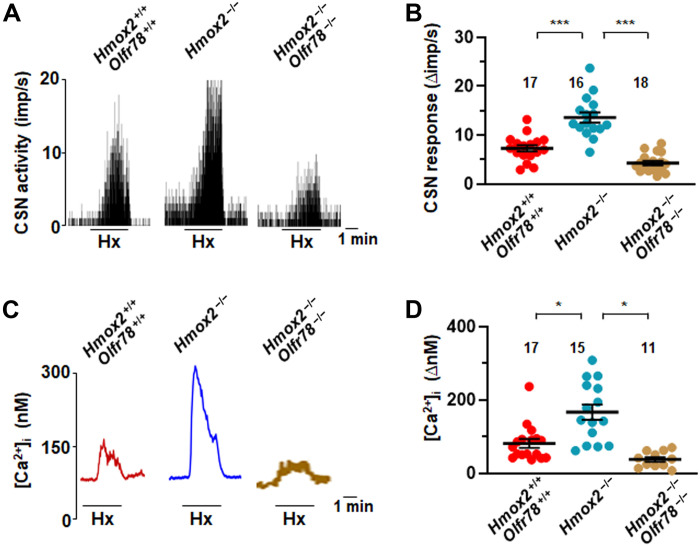
Carotid body and glomus cell responses to hypoxia are impaired in *Hmox-2 ^−/−^ Olfr78*^−/−^ double-mutant mice. CSN and glomus cell [Ca^2+^]_i_ responses to hypoxia in *Hmox-2*/*Olfr78* double mutant compared to *Hmox-2* mutant and WT mice. Example responses of CSN activity in (**A**) and average (means ± SEM) along with individual data of CSN responses to hypoxia in (**B**). Examples of [Ca^2+^]_i_ responses to hypoxia (Hx) in (**C**) and average (means ± SEM) along with individual data of [Ca^2+^]_i_ responses in (**D**). Black bars represent the duration of hypoxia application. *P < 0.05 and ***P < 0.001; one-way ANOVA followed by Holm-Sidak test in (B) and one-way ANOVA on ranks followed by Dunn test in (D).

To establish a role for endogenous H_2_S, we studied carotid bodies from *heme-oxygenase 2* (*Hmox-2*)–null mice, which exhibit higher H_2_S abundance under both basal (normoxia) and hypoxic conditions than WT controls (fig. S10), as reported previously (*23*). We measured CSN activity and glomus cell [Ca^2+^]_i_ responses to hypoxia in *Hmox-2* mutant and *Hmox-2*/*Olfr78* double-mutant mice. Consistent with previous reports (*11*, *23*), basal and hypoxia-evoked CSN activity and glomus cell [Ca^2+^]_i_ responses were enhanced in carotid bodies and cells from *Hmox-2* mutants compared with the responses in WT controls (Fig. 5, A to D). However, the response to hypoxia was abrogated in *Hmox-2*/*Olfr78* double mutants (Fig. 5, A to D), despite the carotid bodies from these mice having increased H_2_S abundance under both normoxia and hypoxia conditions (fig. S10). These results established that Olfr78 is obligatory for CSN and glomus cell activation by endogenous H_2_S.

Systemic application of NaHS stimulated breathing in WT mice but not in *Olfr78* mutants (fig. S11, A to D), consistent with earlier reports showing that carotid body chemo reflex mediates breathing stimulation by H_2_S (*24*, *25*). Collectively, these findings showed that H_2_S activates carotid body through Olfr78 as evidenced by impaired CSN, glomus cell, and breathing responses to H_2_S in *Olfr78* mutant mice.

### Glomus cells are positive for Olfr78 signaling proteins

**Fig. 6.**
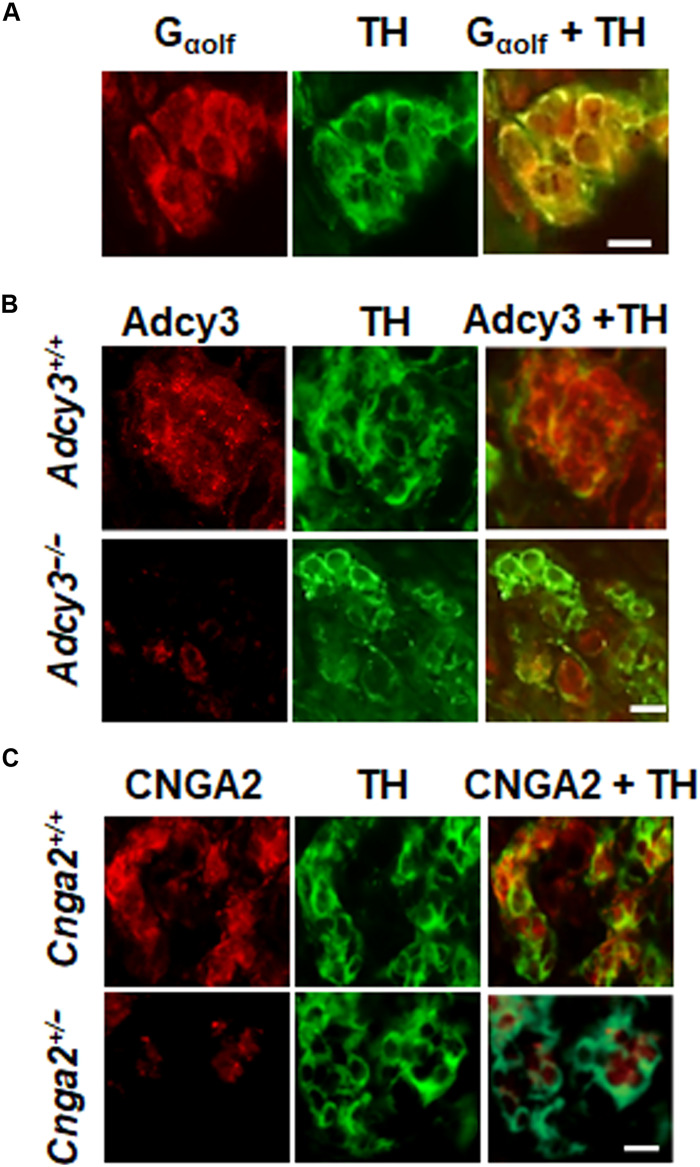
Glomus cells are positive for odorant signaling proteins. (**A** to **C**) Immunocytochemistry of glomus cells for Gα_olf_ in WT (A), *Adcy3* mutant and WT (B), or *Cnga2* mutant and WT (C). Glomus cells were identified by the presence of tyrosine hydroxylase (TH). Scale bars, 10 μm. Example sections were taken from four experiments for each genotype.

Olfr78 belongs to the family of GPCRs detecting odorant stimuli. Odorant receptors are coupled to olfactory G protein G_olf_ (*26*). Carotid body sections were stained with anti-G_olf_ antibody to evaluate the presence of G_olf_ in glomus cells, which are identified by staining with anti–tyrosine hydroxylase (TH) antibody, an established marker of these cells (*1*). Glomus cells of the carotid body were positive for G_olf_, as indicated by colocalization with TH (Fig. 6A). G_olf_ is linked to type III adenylyl cyclase (Adcy3) (*26*). Glomus cells from WT mice are positive for Adcy3, whereas it was absent in mice with floxed *Adcy3* paired with *Th-cre* mice producing cell type–specific knockdown of *Adcy3* in TH-positive glomus cells (Fig. 6B). cAMP opens a cyclic nucleotide–gated channel, Cnga2. We detected Cnga2-like immunoreactivity in TH-positive glomus cells from carotid bodies of WT mice, and this signal was markedly reduced in cells from *Cnga2*^+/−^ mice (Fig. 6C). These results demonstrated that glomus cells are positive for G_olf_, Adcy3, and Cnga2, key molecules associated with odorant receptor signaling.

### Hypoxia and H_2_S increase Adcy3-dependent cAMP

**Fig. 7.**
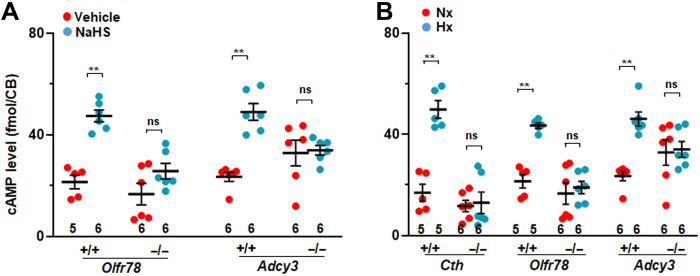
NaHS and hypoxia activate Adcy3-dependent cAMP in carotid bodies. (**A** and **B**) cAMP was measured in carotid bodies from mice of the indicated genotypes after 5-min exposure to NaHS (50 μM) or vehicle or 5-min exposure to hypoxia (Hx; P_O___2__ ~ 40 mmHg) or normoxia (Nx; P_O___2__ ~ 140 mmHg). Data are presented as means ± SEM from the number of mice indicated at the bottom (two carotid bodies were pooled from one mouse for each experiment). **P < 0. 01; ns, *P* > 0.05; two-way ANOVA followed by Holm-Sidak test. CB, carotid body

Adcy3 catalyzes cAMP production. We examined whether NaHS or hypoxia increase Adcy3-dependent cAMP abundance in carotid bodies. NaHS (50 μM) increased cAMP abundance in WT carotid bodies but failed to stimulate an increase in cAMP in carotid bodies from the *Olfr78*-null or *Adcy3* mutant mice (Fig. 7A). We observed that basal cAMP abundance tended to be higher in carotid bodies of *Adcy3* mutant mice (Fig. 7A), likely because of compensatory up-regulation of adenylyl cyclases other than Adcy3 in the carotid body (*27*). Hypoxia (P_O___2__ ~ 40 mmHg) also increased cAMP abundance in carotid bodies from WT mice (Fig. 7B), a finding consistent with earlier studies (*28*), but this effect was absent in mice globally lacking *Cth* or *Olfr78* or lacking *Adcy3* in TH-positive cells (Fig. 7B).

### Carotid body, glomus cell, and breathing responses to hypoxia are impaired in *Adcy3* and *Cgna2* mutants

**Fig. 8.**
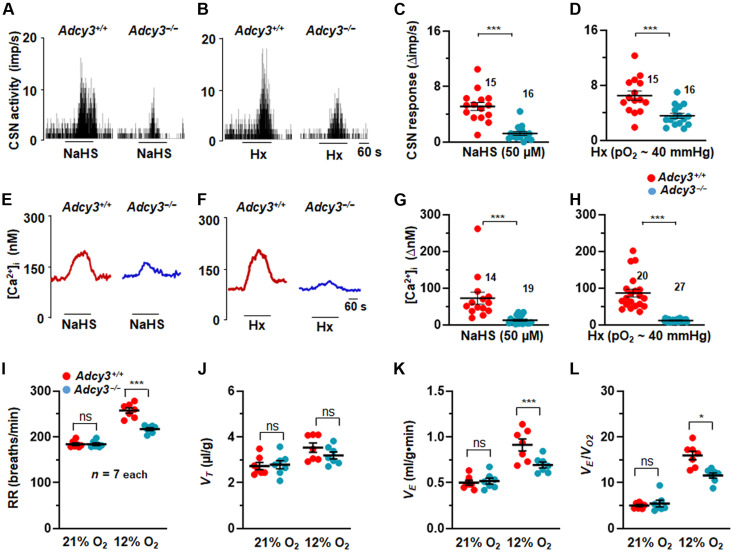
CSN and glomus cell responses to NaHS and hypoxia are impaired in *Adcy3* mutants. (**A** and **B**) Examples of CSN responses to NaHS [50 μM in (A)] or hypoxia (Hx; P_O___2__ ~ 40 mmHg) (B) from WT and *Adcys3* ^−/−^ mice. Black bars represent duration of NaHS and Hx application. Means ± SEM along with individual data (stimulus-baseline activity) are presented for NaHS in (**C**) and for hypoxia in (**D**). (**E** to **H**) [Ca^2+^]_i_ responses to NaHS and Hx in glomus cells. (E and F) Examples of [Ca^2+^]_i_ responses to NaHS (50 μM) (E) and Hx (F), and means ± SEM along with individual data (stimulus-baseline) for NaHS in (G) and hypoxia (H). Numbers indicate the number of cells. (**I** to **L**) Average and individual data of respiratory rate (RR) (breaths/min) (I), tidal volume (*V*_T_, μl/g) (J), minute ventilation (V_E_, ml/g.min) (K), and ratio of minute ventilation/O_2_ consumption (*V*_E_/*V*_O2_) (L). *P < 0.05 and ***P < 0.001; ns, *P* > 0.05; two-way ANOVA with repeated measures followed by Holm-Sidak test.

CSN activity and glomus cell [Ca^2+^]_i_ were monitored to determine the importance of cAMP production by Adcy3 in carotid body responses to NaHS or hypoxia. CSN and glomus cell [Ca^2+^]_i_ responses to NaHS and hypoxia were attenuated in carotid bodies or cells from *Adcy3* mutant mice compared to the responses in WT controls (Fig. 8, A to H). These observations suggested that hypoxia-evoked cAMP production requires Adcy3 activation by H_2_S-Olfr78 interaction and that the increased cAMP contributes to carotid body and glomus cell activation by H_2_S and hypoxia.

Consistent with impaired carotid body response to hypoxia, breathing responses to hypoxia (12% O_2_) were also impaired in *Adcy3* mutants compared to WT control mice (Fig. 8, I to L; fig. S12; and table S1). However, ventilatory responses to 5% CO_2_ were comparable between *Adcy3*-null and WT mice (fig. S13 and table S2).

**Fig. 9.**
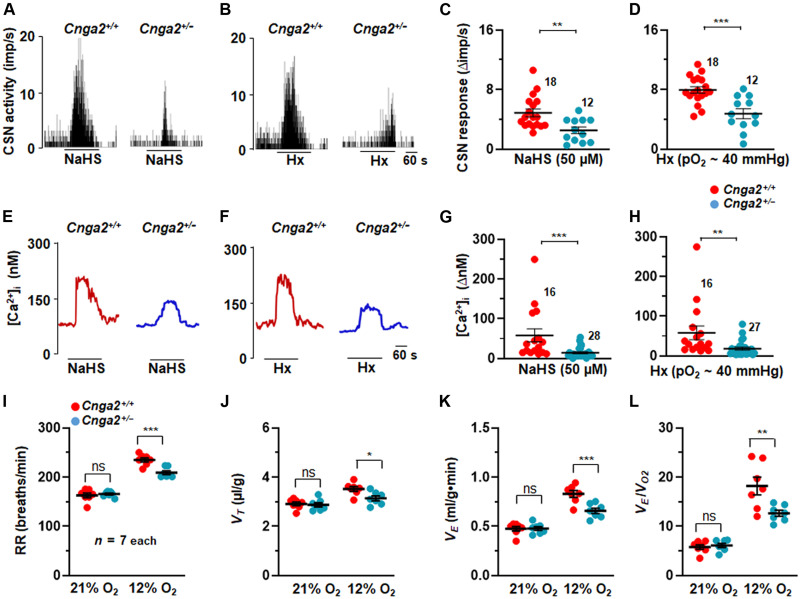
CSN and glomus cell responses to NaHS and hypoxia are impaired in *Cnga2* mutants. (**A** and **B**) Examples of CSN responses to NaHS (50 μM) (A) or hypoxia (Hx; P_O_2__ ~ 40 mmHg) (B). Black bars represent duration of NaHS and Hx application. Means ± SEM along with individual data (stimulus-baseline activity) are presented for NaHS in (**C**) and for hypoxia in (**D**). Numbers represent the number of carotid bodies. (**E** to **H**) [Ca^2+^]_i_ responses to NaHS and Hx in glomus cells. (E and F) Examples of [Ca^2+^]_i_ responses of glomus cells to NaHS (50 μM) (E) and Hx (F), and means ± SEM along with individual data (stimulus-baseline) for NaHS in (G) and hypoxia (H). Numbers indicate the number of cells. (**I** to **L**) Average and individual data of RR (breaths/min) (I), tidal volume (*V*_T_, μl/g) (J), minute ventilation (*V*_E_, ml/g.min) (K), and ratio of minute ventilation/O_2_ consumption (*V*_E_/*V*_O2_) (L). *P < 0.05 and ***P < 0.001; ns, *P* > 0.05; two-way ANOVA with repeated measures followed by Holm-Sidak test.

cAMP elevation by olfactory receptors opens a cyclic nucleotide–gated channel, Cnga2, and the ensuing influx of cations stimulate olfactory nerve action potentials (*29*). The role of Cnga2 in the carotid bodies was evaluated by measuring CSN activity and glomus cell [Ca^2+^]_i_ responses to NaHS and hypoxia in WT and in *Cgna2* partially deficient mice (*Cgna2*^+/−^). We used mice with partial deficiency of *Cnga2* (*Cnga2*^+/−^) because of poor survival of *Cgna2* homozygous mice. Despite partial deficiency of *Cnga2*, CSN and [Ca^2+^]_i_ responses to NaHS and hypoxia were markedly attenuated in carotid bodies and cells from *Cnga2*^+/−^ mice compared to WT controls (Fig. 9, A to H). However, the CSN response to KCl, a nonselective activator of CSN, was unaltered in *Cnga2*^+/−^ mice (fig. S14, A and B), indicating that the nerves were functional. Forskolin, which increases cAMP, potentiated CSN and glomus cell [Ca^2+^]_i_ responses to hypoxia in WT but not in the *Cnga2*^+/−^ mutants (fig. S15). Likewise, 8-bromo cAMP (8-Br-cAMP), a membrane-permeable analog of cAMP also enhanced CSN responses to NaHS and hypoxia in WT mice but not in *Cnga2*^+/−^ mice (fig. S16, A to D). In contrast, 8-bromo cyclic guanosine monophosphate (8-Br-cGMP) reduced CSN responses to hypoxia (fig. S17, A and B).

*Cnga2*^+/−^ mice manifested an attenuated breathing response to hypoxia (12% O_2_) (Fig. 9, I to L, and fig. S18), whereas changes in VO_2_ and VCO_2_ are comparable between WT and *Cnga2* mutants (table S3). However, breathing responses to 5% CO_2_ are comparable between WT and *Cnga2* mutants (fig. S19 and table S4).

## DISCUSSION

Previous studies suggested that CSE-derived H_2_S is an important mediator of carotid body CSN activation by acute hypoxia (*4*, *23*, *30*). However, mechanism(s) linking H_2_S to the CSN excitation have not been examined. Our results establish that (i) NaHS increases persulfidation labeling in WT carotid bodies but not in Olfr78 mutants and activates Olfr78 as evidenced by increased cAMP fluorescence in HEK293 cells expressing Olfr78 in a dose-dependent manner, as well as persulfidates Cys^240^ of Olfr78 protein, and (ii) CSN and glomus cell activation by NaHS and hypoxia use G protein–coupled odorant receptor signaling.

Although many olfactory receptors do not traffic to the cell membrane when expressed in cultured cells, Olfr78 trafficked to the cell membrane when expressed in HEK293 cells, allowing assessing the direct effect of NaHS on Olfr78. Odorant receptor activation is often measured by cAMP concentrations indirectly by a luciferase assay under the control of cAMP-responsive element (*12*, *13*) or by a genetically encoded fluorescent cAMP biosensor (*16*). The cAMP biosensor approach revealed that NaHS leads to a rapid and short-lived activation of Olfr78, consistent with short half-life of H_2_S as reported earlier (*14*, *15*). Unlike cells expressing Olfr78, NaHS had no effect on cells expressing odorant receptor unrelated to Olfr78. The effects of NaHS appear selective to Olfr78 because H_2_S was ineffective in activating odorant receptor OR2T11, which detects sulfur odor (*31*). Acetate activated Olfr78, consistent with an earlier study (*12*). However, NaHS appears to be more potent than acetate with an EC_50_ of millimolar concentration compared to EC_50_ of 25 μM NaHS.

How might NaHS activate Olfr78? Recent studies suggest that persulfidation is an important mechanism underlying the biological actions of H_2_S (*17*, *32*). The recently described dimedone switch method (*17*) enabled us to demonstrate persulfidation of Olfr78 by NaHS in the carotid body glomus cells visualized by microscopy. Hypoxia, which elevates H_2_S abundance in the carotid body (*4*), increased persulfidation signal in the glomus cells, and this effect was absent in mice lacking *Cth*, which encode CSE, a major H_2_S synthesizing enzyme in the carotid body or carotid bodies of Olfr78 mutant mice.

Microscopy with the dimedone switch approach cannot identify specific Cys residues affected by NaHS. The alpha fold model of Olfr78 indicated Cys^96^, Cys^240^, and Cys^310^ as potential targets of NaHS. MS revealed a time-dependent increase in Cys^240^ persulfidation by NaHS. Substituting Cys^240^ with alanine blocked activation of Olfr78 by H_2_S and impaired trafficking of the mutant protein to the cell surface, further confirming the prediction of the alpha fold model of that Cys^240^ location near the cell membrane. NaHS-evoked Cys^240^ persulfidation was markedly reduced in cells treated with inhibitors of H_2_S production (fig. S4). On the other hand, substituting Cys^96^ or Cys^310^ with alanine had no consistent effect on Olfr78 activation measured by cAMP fluorescence. These results suggested that carotid body responses to NaHS and hypoxia involve redox modification of Cys^240^ in the Olf78 protein.

Jiang *et al.* (*19*) reported that H_2_S donors inhibit the mitochondrial ETC and increases ROS abundance in pluripotent stem cells. A recent study reported that functional mitochondrial ETC is required for glomus cell responses to hypoxia (P_O_2__ ~ 10 to 15 mmHg) (*33*). It can be argued that carotid body response to NaHS might also involve mitochondrial ETC and ROS production. We measured MMP of glomus cells as an index of change in mitochondrial ETC. Whereas 1 and 50 μM NaHS had no effect on the MMP of glomus cells, 300 μM NaHS did increase MMP (fig. S8). In addition, 300 μM NaHS, which increased MMP, produced only a brief and small CSN activation followed by inhibition. On the other hand, 50 μM NaHS, which had no effect on MMP, produced a robust CSN activation (fig. S8), demonstrating divergence between MMP and CSN response to NaHS. Moreover, treating carotid bodies with Mito-TEMPO, a scavenger of mitochondrial ROS, had no effect on CSN activation by either NaHS or hypoxia (fig. S7). Olfr78 mutants exhibited impaired CSN and glomus cell responses to NaHS and hypoxia (Fig. 4), demonstrating that the effects of NaHS primarily involve Olfr78 without affecting mitochondria in glomus cells. The unaltered CSN response to NaCN in Olfr78 mutants indicates that Olfr78 signaling bypass the complex IV of the mitochondria.

Glomus cells are positive for G_olf_, Adcy3, and Cnga2, representing key molecules implicated in GPCR signaling. Unavailability of G_olf_-null mice precluded assessing its impact on carotid body activation either by NaHS or hypoxia. Notwithstanding this limitation, we found severely impaired CSN, as well as glomus cell responses to NaHS and hypoxia in *Adcy3* and *Cnga2* mutants. Consistent with carotid body responses to hypoxia, breathing responses to low O_2_ were equally impaired in *Adcy3* and *Cnga2* mutants. Increased cAMP abundance observed with hypoxia is consistent with an earlier study showing that hypoxia increases cAMP, and phosphodiesterase-4 inhibitor potentiates the carotid body cAMP response to hypoxia (*34*).

**Fig. 10.**
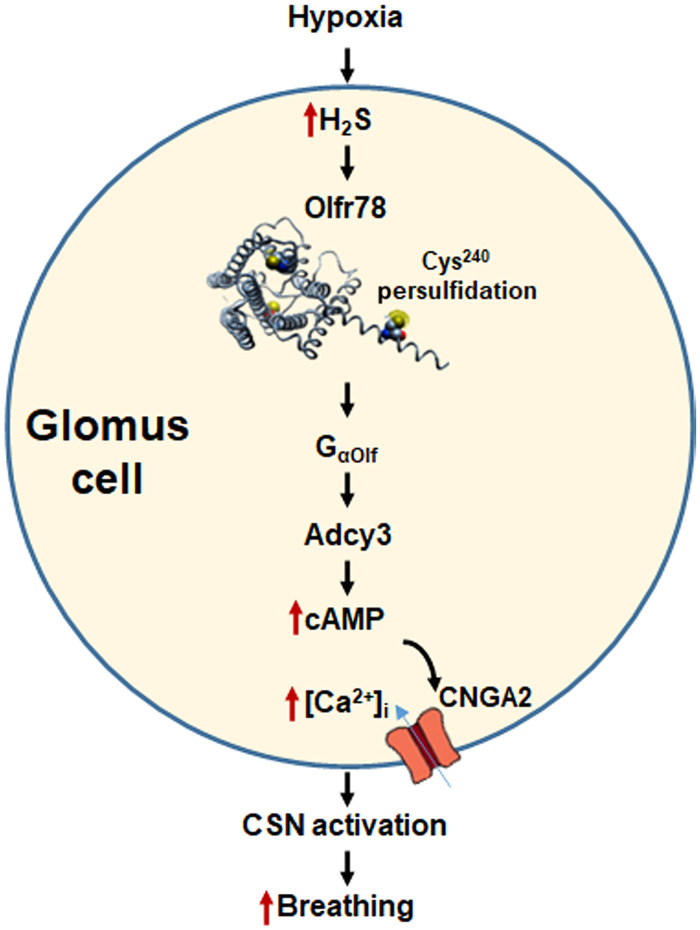
H_2_S-Olfr78 signaling in carotid body response to hypoxia. Abbreviations: H_2_S, hydrogen sulfide; Olfr78, olfactory receptor 78; Cys^240^, persulfidated Cys residue in Olfr78; G_olf_, G protein coupled to Olfr78; Adcy3, adenylate cyclase 3; cAMP, cyclic adenosine monophosphate; [Ca^2+^]_i_, intracellular calcium concentration; Cnaga2, cyclic nucleotide–gated channel α2; CSN activation, carotid body sensory nerve activation.

Both cAMP and cGMP can activate Cnga2. The following observations demonstrate that cAMP rather than cGMP contribute to Cnga2 activation in the carotid body: (i) forskolin, which increases cAMP by activating adenylyl cyclases (*35*), potentiated CSN and glomus cell response to hypoxia, and these effects are absent in *Cnga2^+/−^* mice; (ii) 8-Br-cAMP also enhanced CSN, glomus cell response to hypoxia, and NaHS, and these effects are markedly impaired or absent in *Cnga2^+/−^* mice; (iii) in sharp contrast, 8-Br-cGMP inhibited CSN activation by hypoxia. Studies on CSN and breathing responses to either NaHS or hypoxia are attenuated on average by 55 to 60% in Adcy3 mutants. It is likely that the residual breathing responses involve compensatory up-regulation of adenyl cyclases other than Adcy3. Nonetheless, these findings together with impaired breathing response to hypoxia but not to CO_2_ in *Adcy3* and *Cnga2* mutants provide evidence for participation of Olfr78-dependent activation of GPCR signaling in CSN and glomus cell activation NaHS and hypoxia. Signaling pathways associated with H_2_S/Olfr78 in hypoxic sensing by the carotid body delineated in the current study are summarized in Fig. 10.

Studies with Adcy3 and Cnga2 mutants reveal remarkable similarities between hypoxic sensing by the carotid body and odorant detection by olfactory receptors. Recently, it was reported hypoxia and H_2_S activate type B sensory neurons and evoke Ca^2+^ influx of mouse olfactory epithelium (*36*, *37*). Response to hypoxia involves soluble guanylate cyclase (Gucy 1b2) and transient receptor potential (Trpc2) channels (*36*). Similarities between sensory neurons of olfactory epithelium by odorant stimuli and carotid body excitation by hypoxia likely represent examples of nature repurposing signaling machinery for sensing distinct modalities of chemical stimuli (hypoxia versus odorant stimuli). Unlike the olfactory system, hypoxic sensing by carotid body upstream to Olfr78 involves O_2_-dependent enzymatic generation of CO and H_2_S as described previously (*23*). Unlike other gaseous transmitters (e.g., NO and CO), H_2_S appears to use GPCR signaling similar to other transmitters such as catecholamines in the nervous system.

In addition to maintaining homeostasis under hypoxia, carotid body chemo reflex plays an important role in pathophysiology associated with obstructive sleep apnea, which is a highly prevalent respiratory disorder, as well as cardiorespiratory adaptations to hypobaric hypoxia such as experienced at high altitude. Earlier studies reported that carotid body activation by H_2_S signaling plays an important role in mediating hypertension in a rodent model of sleep apnea (*38*) and carotid body–dependent cardiorespiratory adaptations to hypobaric hypoxia (*39*). Whether chronic intermittent hypoxia and hypobaric hypoxia affect GPCR signaling associated with H_2_S-Olfr78 interaction in the carotid body remains to be investigated.

### Limitations

The following are the limitations of the current study. Unstimulated GPCRs exhibit an inactive structural conformation and attain active structural conformation following stimulation by ligands. Whether activation of Olfr78 by H_2_S or hypoxia involves Olfr78 active structural conformation remains to be investigated. Currently used techniques for measuring H_2_S concentrations are inadequate for measuring actual H_2_S concentrations near glomus cells. Therefore, the absence of measurements of actual concentrations of H_2_S near glomus cells is a technical limitation of the current study. Although findings with the *Cnga2*^+/−^ mice suggested a role for these channels in CSN excitation by H_2_S and hypoxia, future studies with direct measurements of Cnga2 channel activity are necessary to establish a role of these channels in carotid body responses to H_2_S and hypoxia.

## MATERIALS AND METHODS

### General preparation

Experimental protocols were approved by the Institutional Animal Care and Use Committee of the University of Chicago (protocol no. ACUP 71811, approved on 27 February 2019). Studies were performed on age- and gender-matched (both males and females) adult (3 to 5 months old) WT, Olfr78-null (from J. Pluznick, the Johns Hopkins University) (*40*), HO-2 (*Hmox-2*)–null (from S. H. Snyder, the Johns Hopkins University; initially generated by S. Tonegawa) (*41*), *TH-Cre/Adcy3 ^f/f^* (*Adcy3 ^f/f^* from X. Chen, the University of New Hampshire and D. R. Storm, the University of Washington) (*42*), *Cth*-null (from R. Wang, York University Toronto, Ontario, Canada) (*4*), and *Cnga2*^+/−^ (Jackson Laboratory, stock no. 002905) mice. Olfr78-null mice were initially generated by Bozza *et al.* (*40*) and subsequently backcrossed with C57BL/6 mice by J. Pluznick. *Hmox-2*/*Olfr78* double-null mice were generated by crossing *Hmox-2*– and *Olfr78*-null mice, as well as *TH-Cre/Adcy3 ^f/f^* mice by crossing *TH-Cre* (Jackson Laboratory, stock no. 008601) and *Adcy3 ^f/f^* mice at the University of Chicago. All mice were on C57BL/6 background. Experiments were performed by individuals blinded to the genotype. The following chemicals were used: NaHS (Sigma-Aldrich, MO, USA), 8-Br-cAMP and 8-Br-cGMP (Cayman Chemical), and forskolin (Cayman Chemical).

### Heterologous expression of Olfr78 in HEK293 cells and immunocytochemistry

The protocols for heterologous expression of Olfr78 in HEK293 cells are essentially the same as described in an earlier study (*12*, *13*). Briefly, HEK293 cells were plated on a 96-well plate (~2 × 10^4^ cells/100 μl per well) and cultured with Dulbecco’s modified Eagle’s medium (DMEM) at 37°C and 5% CO_2_ overnight and transfected with flag-tagged Olfr78 (mouse), OR51E2 (human), or mOR-EG (mouse) plasmids using a TransIT-2020 (Mirus) transfection reagent. For surface staining, transfected cells plated on poly-lysine coverslips were washed with phosphate-buffered saline (PBS; pH 7.4) containing 0.1% bovine serum albumin (BSA) for 30 min 4°C and incubated with anti-flag polyclonal antibody (1/200 dilution; Sigma-Aldrich, #F7425) for 1 hour at 4°C. Cells were fixed with 4% formaldehyde at room temperature (RT) for 30 min followed by incubation in permeabilization buffer [0.3% Triton X-100 and 1% BSA in PBS (pH 7.4)] for 15 min at RT. Coverslips were incubated with blocking buffer [1% BSA and 0.2% milk in tris-buffered saline (pH 7.4)] for 30 min at RT, and M2 monoclonal flag antibody (1/200 dilution; Sigma-Aldrich, F1804) was added for 1 hour at RT (or 4°C overnight). After three washes with PBS, coverslips were incubated with secondary antibodies [fluorescein isothiocyanate (FITC) 488 or Alexa Fluor 555; Life Sciences) in blocking buffer for 45 min at RT and mounted in Vectashield with 4′,6-diamidino-2-phenylindole (DAPI) (Vector Laboratories *H-1200).

### cAMP biosensor

A genetically encoded fluorescent cAMP sensor, green upward (Montana Molecular, #U0205G), was used to track intracellular cAMP abundance. HEK293 cells were cultured as described above and were grown on glass coverslips. The cAMP reporter was prepared as follows: 1.6 ml of the sensor was combined with 48 μl of sodium butyrate and 2.35 ml of culture medium. Each culture dish had 1.6 ml of medium to which 0.8 ml of the cAMP reporter mixture was added. Cells were incubated for 24 hours in this solution at 37°C in 5% CO_2_ before addition of a stimulus.

### Measurement of cAMP fluorescence in HEK293 cells

In initial experiments, cells expressing the cAMP reporter were imaged on a Leica TCS SP5 II AOBS confocal system on a DMI6000 microscope using a 40× numerical aperture 1.25 oil objective, 488-nm laser excitation, 2–Airy unit pinhole, HyD detector with 495 to 540 emission range, and scanned at 8 kHz (line average 16). Transmitted differential interference contrast images were collected coincident with fluorescence. Time-lapse capture of four adjacent fields of view was automatically collected and stitched using LASAF software. The reporter signal was calculated as *F* over *F*_0_ for cells positive for the probe. Pseudo-color images were created using FIJI software.

For assessing the concentration-dependent effects of H_2_S, cAMP fluorescence was measured using a microplate reader by setting the wavelength of excitation at 495 nm and emission at 540 nm (Synergy H1, Biotek, Winooski, VT). After collecting two baseline readings of cAMP fluorescence, 10 μl of NaHS with various concentrations, 15 mM sodium acetate (NaAce, included in the kit), or 5 μM isoproterenol (ISOP, in the kit) was added, and cAMP fluorescence intensity was recorded every minute for 8 min. Changes in cAMP fluorescence intensity in response to a given stimulus were quantified using the following equation: Δ*F* = (*F* − *F*_0_)/*F*_0_, where *F* is the reading of cAMP fluorescence intensity after the stimulus was added, *F*_0_ is the reading from the same well before the stimulus was applied, and Δ*F* represents changes in cAMP fluorescence intensity.

### Persulfidation detection in the carotid body by microscopy

Procedures for detecting persulfidation in carotid body sections are essentially the same as described earlier (*18*). Briefly, carotid bifurcations were removed from anesthetized mice and were treated with room air (normoxia; P_O_2__ ~ 140 mmHg), hypoxia (medium P_O_2__ ~ 40 mmHg for 5 min), NaHS (50 μM for 5 min), or vehicle at 37°C. Tissues were fixed in 100% methanol at −20°C for 4 hours. The methanol-fixed tissue was placed in 30% sucrose-PBS overnight at 4°C and then frozen in Tissue-Tek (OCT; VWR Scientific), serially sectioned at 8 μm (Leica, CM1900), and mounted on collagen-coated coverslips. Sections were treated with acetone for 5 min at −20°C, followed by three washes with PBS for 5 min at 37°C. After incubation with 1 mM NBF-Cl (Sigma-Aldrich, #163260) in PBS for 2 hours at 37°C, sections were washed extensively with PBS at RT, left overnight, and then washed at 4°C with agitation. Sections were then incubated with 10 μM DAz-2:Cy-5 mix (DAz-2, Cayman Chemical, no. 13382; Cy-5, Lumiprobe, #B30B0) in PBS for 30 min at 37°C. For negative control, sections were incubated with 10 mM DAz-2:Cy-5 click mix prepared without DAz-2. Subsequently, sections were washed with PBS overnight with agitation, protected from light, followed by three washes with 100% methanol for 10 min at RT and five washes with PBS for 5 min. Sections were mounted in Vectashield with DAPI (Vector Laboratories *H-1200) and visualized using an all-in-one fluorescent microscope (BZ-X810; Keyence Corp. of America, Itasca, IL). Sections were examined at 488 nm (for NBF adducts) and 633 nm (Cy5 for PSSH).

### MS/MS analysis

HEK293 cells were plated on a 96-well plate (~2 × 10^4^ cells/100 μl per well) and cultured with DMEM at 37°C and 5% CO_2_ overnight and transfected with flag-tagged Olfr78 (mouse) using a TransIT-2020 (Mirus) transfection reagent. Cells were treated with 25 μM NaHS for 5 or 10 min. For inhibition of H_2_S production, cells were treated with a mix of 1 mM *O*-(carboxymethyl)hydroxylamine hemihydrochloride and d,l-propargylglycine for 30 min. Frozen cell pellets were lysed in HEN buffer [50 mM Hepes, 1 mM EDTA, 0.1 mM neocuproine, 1% NP-40, and 2% SDS (pH 7.4)] supplemented with 1% protease inhibitor and 20 mM IAM using a Bioruptor (Diagenode). Lysates were incubated at +37°C for 2 hours and precipitated with methanol/chloroform precipitation [H_2_O/MeOH/CHCl_3_, 4/4/1 (v/v/v)]. Pellets were resuspended in PBS, and protein concentration was adjusted to 1 mg/ml. A total of 600 μg of each pellet was incubated with anti-flag antibodies coupled to magnetic beads (#M8823, Sigma-Aldrich) on Ferris Wheel. Using a magnetic rack, the beads were sequentially washed three times with PBS containing 0.01% Tween-20 and three times with PBS. Beads were then resuspended in 100 μl of 50 mM ammonium bicarbonate, 1 mM CaCl_2_, and trypsin (0.025 μg/μl; Promega). Samples were incubated for 16 hours at 37°C. Supernatants were then collected. For double digestion, chymotrypsin was added at 0.0125 μg/μl and incubated at 30°C for 10 hours. Samples were then desalted using HLB-SPE columns (Sigma-Aldrich) and dried. Peptides were dissolved in 0.1% trifluoroacetic acid before being analyzed by high-resolution liquid chromatography–MS/MS using an Ultimate 3000 Nano Ultra High-Pressure Chromatography (UPLC) system (Thermo Fisher Scientific) coupled with an Orbitrap Eclipse Tribrid mass spectrometer via an EASY-spray (Thermo Fisher Scientific). Peptide separation was carried out with an Acclaim PepMap 100 C18 column (Thermo Fisher Scientific) using a 120-min gradient (0 min, 3% B; 120 min, 35%: 84% acetonitrile and 0.1% formic acid) at a flow rate of 250 nl/min. The Orbitrap Eclipse was operated in a DDA mode, and MS1 survey scans were acquired from mass/charge ratio (*m*/*z*) 300 to 1800 at a resolution of 120,000 using the Orbitrap mode. The most intense ions were isolated for 3 s with a 1 *m*/*z* of window and then fragmented by high-energy collision-induced dissociation with a normalized collision energy of 32%, considering a dynamic exclusion of 20 s. MS/MS spectra were recorded using Normal Iontrap mode. Data evaluation was performed with PEAKS online software using 10 parts per million for precursor mass tolerance, 0.5 Da for fragment mass tolerance, and a maximum of three missed cleavages. Acetylation (N-term); carbamidomethylation (C; +57.02 Da); oxidation (M); and persulfidation, i.e., S-S-IAM (C; +89.08 Da) were used as variable modifications.

### Measurement of CSN activity

CSN activity was recorded ex vivo as previously described (*10*, *38*). Briefly, carotid bodies along with the sinus nerves were harvested from anesthetized [urethane; 1.2 g/kg, intraperitoneally (ip)] mice, placed in a 250-μl recording chamber and superfused with warm physiological saline (35°C) at a rate of 3 ml/min. The composition of the physiological saline was as follows: 125 mM NaCl, 5 mM KCl, 1.8 mM CaCl_2_, 2 mM MgSO_4_, 1.2 mM NaH_2_PO_4_, 25 mM NaHCO_3_, 10 mM d-glucose, and 5 mM sucrose. The solution was bubbled with 21% O_2_/5% CO_2_. Hypoxic challenges were achieved by switching the perfusate to saline equilibrated with desired amounts of O_2_. Oxygen in the saline was continuously monitored using a platinum electrode placed next to the carotid body, which was connected to a polarographic amplifier (Model 1900, A-M Systems, Sequim, WA). To facilitate recording of action potentials, the sinus nerve was treated with 0.1% collagenase for 5 min. Action potentials (one to three active units) were recorded from one of the nerve bundles with a suction electrode, filtered (bandpass, 100 to 3000 Hz), amplified (P511K; Grass Technologies, Natus Neurology, Middleton, WI), collected (sampling rate of 20 kHz), and stored in a computer with a data acquisition system (Power Lab/8P, AD Instruments, Colorado Springs, CO). “Single” units were sorted on the basis of the shape, height, and duration of the individual action potentials using the spike discrimination module. For assessing the CSN response to CO_2_, a bicarbonate-buffered medium was equilibrated with either 90% O_2_ + 5% CO_2_ (baseline) or 90% O_2_ + 10% CO_2_, and solution CO_2_ amounts were determined using a blood gas analyzer (ABL-80, Radiometer, Copenhagen, Denmark). To examine the effect of 20 mM K^+^ on CSN activity, equal molar of NaCl in the saline was replaced with KCl. NaHS (Sigma-Aldrich, St. Louis, MO) or forskolin (Cayman Chemical, Ann Arbor, MI) was added at the indicated concentrations to the saline.

### Measurement of efferent phrenic nerve activity

Mice were anesthetized with intraperitoneal injections of urethane (1.2 g/kg). Supplemental doses (10% of the initial dose of anesthetic) were given when corneal reflexes or responses to toe pinches were observed. Animals were placed on a warm surgical board, and tracheotomy was performed through a midline neck incision. The trachea was cannulated, and mice were allowed to breathe spontaneously. Core body temperature was monitored using a rectal thermistor probe and maintained at 38°C using a heating pad. The phrenic nerve was isolated unilaterally at the level of the C3 and C4 spinal segments, cut distally, and placed on bipolar stainless-steel electrodes. Integrated efferent phrenic nerve activity was monitored as an index of respiratory neuronal output. The electrical activity was filtered (bandpass, 30 to 10,000 Hz), amplified (P511K, Grass Technologies), collected (sampling rate of 10 kHz), and stored in a computer for further analysis (PowerLab/8P). Phrenic nerve activity [bursts per minute; an index of respiratory rate (RR)], tidal phrenic nerve activity [in arbitrary units (a.u.)], and minute neuronal respiration (MNR = RR × tidal phrenic nerve activity) were analyzed. The effects of NaHS on efferent phrenic nerve activity were tested by administering increasing doses of freshly prepared NaHS (1, 10, 30, and 60 μg/kg) through a catheter (PE-10) inserted into the right external jugular vein. NaHS was freshly prepared in warm saline before each experiment. The volume of administration was 100 μl for each dose. At the end of the experiment, mice were euthanized by overdose of urethane (>3.6 g/kg, ip).

### Measurements of ventilation and metabolic variables

In unanesthetized animals, ventilation was monitored using whole body plethysmograph as previously described (*10*). Briefly, animals were placed in a Lucite chamber containing an inlet port for gas administration and were allowed to acclimate for 1 hour in room air. The chamber was connected to a high-gain differential pressure transducer, and breathing signals were converted and amplified (Buxco, DSI, St. Paul, MN), recorded, and stored in a computer via an A/D translation board (PowerLab/8P) for further analysis. Oxygen consumption (V_O_2__) and CO_2_ production (V_CO_2__) were determined as described (*10*). Sighs, sniffs, and movement-induced changes in breathing and metabolic variables were monitored and excluded in the analysis. All recordings were made at an ambient temperature of 25 ± 1°C.

### Primary glomus cell culture

Preparation of primary cultures of glomus cells was performed as described previously (*10*, *30*). Briefly, carotid bodies were harvested from mice anesthetized with urethane (1.2 g/kg, ip), and glomus cells were dissociated using a mixture of collagenase P (2 mg/ml; Roche Applied Science, Indianapolis, IN), deoxyribonuclase (DNase) (15 μg/ml; Sigma-Aldrich), and BSA (3 mg/ml; Sigma-Aldrich) at 37°C for 20 min, followed by a 15-min incubation in Locke’s buffer containing DNase (30 μg/ml). Cells were plated on collagen (type VII; Sigma-Aldrich)–coated coverslips and maintained at 37°C in a 7% CO_2_ + 20% O_2_ incubator for 12 to 18 h. The growth medium consisted of DMEM/F-12 medium (Invitrogen, Thermo Fisher Scientific, Waltham, MA), supplemented with 1% fetal bovine serum, insulin-transferrin-selenium (ITS-X; Invitrogen), and 1% penicillin-streptomycin-glutamine mixture (Invitrogen).

### Measurements of [Ca^2+^]_i_ and MMP

#### Measurements of [Ca^2+^]_i_

Glomus cells were incubated in Hanks’ balanced salt solution (HBSS; Thermo Fisher Scientific) with 2 μM fura-2 AM (Biotium Inc., Fremont, CA) and BSA (1 mg/ml) for 30 min and then washed in a fura-2–free solution for 30 min at 37°C. The coverslip was transferred to an experimental chamber for determining the changes in [Ca^2+^]_i_. Background fluorescence was obtained at 340- and 380-nm wavelengths from an area of the coverslip devoid of cells. On each coverslip, glomus cells were identified by their characteristic clustering, and individual cells were imaged with a Leica microscope equipped with a Hamamatsu camera (model C11440) using the software HC Image (version 4.5.1.3). Image pairs (one at 340-nm and the other at 380-nm wavelength) were obtained every 2 s by averaging 16 frames at each wavelength. Data were continuously collected throughout the experiment. Background fluorescence was subtracted from the cell data obtained at the individual wavelengths. Fluorescence intensity of the image obtained at 340 nm was divided by that at 380 nm to produce a ratiometric image. Ratios were converted to free [Ca^2+^]_i_ using calibration curves constructed in vitro by adding fura-2 (50 μM free acid) to solutions containing known concentrations of Ca^2+^ (0 to 2000 nM). The recording chamber was continually irrigated with warm physiological saline (31°C) from gravity-fed reservoirs. The composition of the saline was the same as that used for recording CSN activity.

#### Measurements of MMP

The protocol for measuring MMP was the same as the measurement of [Ca^2+^]_i_ except for a few modifications. Briefly, glomus cells were incubated in HBSS with rhodamine 123 (10 μg/ml; Cayman Chemical, #16672) and BSA (1 mg/ml) for 15 min and then washed in a rhodamine 123–free solution for 15 min at 37°C. Rhodamine 123 fluorescence was excited at 500 nm and measured at 535 nm. Background fluorescence was determined from areas devoid of cells and subtracted from the fluorescence intensity with glomus cells. Changes in MMP are presented as a percentage change in fluorescence intensity from the baseline level and normalized as a percentage of the response evoked by 1 min of application of 5 μM FCCP (Cayman Chemical, #15218) and oligomycin complex (20 μg/ml; Cayman Chemical, #11341) for 3 min at the end of each experiment. Only the cells exhibiting more than 20% changes in MMP in response to FCCP were included for analysis.

### Measurements of cAMP in carotid bodies

Carotid bodies harvested from anesthetized (urethane; 1.2 g/kg, ip) mice were placed in a 250-μl chamber and superfused with warm physiological saline (35°C) at a rate of 3 ml/min. The composition of the saline was the same as that used for recording CSN activity with the addition of 100 μM 3-isobutyl-1-methylxanthine (Sigma-Aldrich). In the experiments assessing the effects of hypoxia, carotid bodies were initially superfused with normoxic saline [partial pressure of oxygen (P_O_2__) ~ 140 mmHg] for 10 min followed by hypoxic medium (P_O_2__ ~ 40 mmHg) for 5 min. To test the effects of H_2_S, control experiments were performed by superfusing carotid bodies with normoxic medium (P_O_2__ ~ 140 mmHg) for 10 min followed by addition of physiological saline (vehicle) for 5 min. The protocols were repeated by replacing NaHS (50 μM) for vehicle. After each treatment, the carotid bodies were removed quickly from the chamber, frozen with liquid nitrogen, and kept at −80°C until further experiments. Carotid body cAMP abundance was measured using a cAMP enzyme-linked immunosorbent assay kit (STA-501, Cell Biolabs Inc., San Diego, CA) according to the manufacturer’s instructions. In each experiment, two carotid bodies were pooled, homogenized in 100 μl of lysis buffer on ice for 30 min, and centrifuged for 5 min (16,000*g*). The supernatant (50 μl) was added to a well of a 96-well plate. Diluted peroxidase cAMP tracer conjugate (25 μl) and diluted rabbit anti-cAMP polyclonal antibody (50 μl) were added to each well, and the plate was incubated for 30 min at RT. After five washes with wash buffer, each well was incubated with 100 μl of chemiluminescent reagent for 5 min, and luminescence was read with a microplate luminometer. With each experiment, a corresponding standard curve was generated with cAMP standards, and this standard curve was used to calculate cAMP content in the carotid bodies. The cAMP content is expressed as femtomoles of cAMP per carotid body. The sensitivity of the assay was 1 pM cAMP.

### Immunohistochemistry

Anesthetized mice were perfused intracardially with heparinized PBS (pH 7.4) at a rate of 10 ml/min for 10 min followed by buffered formaldehyde (4% formalin; Thermo Fisher Scientific) for 30 min. Carotid bifurcations were removed and placed in 4°C 4% paraformaldehyde-PBS for 1 hour. After washing with PBS, carotid bifurcations were placed in 30% sucrose-PBS at 4°C for 24 hours. Specimens were frozen in Tissue-Tek (OCT; VWR Scientific), serially sectioned at 8 μm (Leica CM1900), and mounted on collagen-coated coverslips. Sections were blocked in PBS containing 1% normal goat serum and 0.2% Triton X-100 and then incubated with polyclonal rabbit anti-G_olf_ antibody (dilution 1:100; MyBioSource, no. MBS9407427), polyclonal rabbit anti-AC3 antibody (dilution 1:500; #AAR-043, Alomone), polyclonal rabbit anti-CNGA2 antibody (dilution 1:200; #APC-045, Alomone), or monoclonal mouse anti-TH antibody (1:1000; Sigma-Aldrich, #T1299), followed by five washes with PBS containing 0.05% Triton X-100. Antibody binding was detected using Texas Red–conjugated goat anti-mouse immunoglobulin G (IgG) or FITC-conjugated goat anti-rabbit IgG (Molecular Probes) diluted 1:250 in PBS containing 1% normal goat serum and 0.2% Triton X-100 (1 hour at 37°C) and washed with PBS containing 0.05% Triton X-100 (five times). Sections were mounted in Vectashield with DAPI (Vector Laboratories *H-1200) and visualized using a fluorescent microscope (Eclipse E600; Nikon).

### Measurements of H_2_S

H_2_S production in the carotid body was assayed as described (*4*, *23*). Briefly, carotid bodies were pooled (six carotid bodies per experiment), and tissue homogenates were prepared in 100 mM potassium phosphate buffer (pH 7.4). The enzyme reaction was carried out in sealed tubes flushed with different levels of O_2_─N_2_ gas mixtures. The P_O_2__ of the reaction medium was determined using a blood gas analyzer (ABL5). The assay mixture in a total volume of 500 μl contained (in final concentration) 800 μM l-cysteine, 80 μM pyridoxal 5′-phosphate, 100 mM potassium phosphate buffer (pH 7.4), and tissue homogenate (2 μg of protein). The reaction mixture was incubated at 37°C for 1 hour, and at the end of the reaction, alkaline zinc acetate [1% (w/v); 250 μl] and trichloroacetic acid [10% (v/v)] were added sequentially to trap H_2_S generated and to stop the reaction, respectively. The zinc sulfide formed was reacted sequentially with acidic *N*,*N*-dimethyl-*p*-phenylenediamine sulfate (20 μM) and ferric chloride (30 μM), and the absorbance was measured at 670 nm using a microplate reader. A standard curve relating the concentration of Na_2_S and absorbance was used to calculate H_2_S concentration and expressed as nanomoles of H_2_S formed per hour per milligram of protein (*4*).

### Statistical analysis of the data

CSN activity (discharge from single units) was averaged for 3 min before hypoxic challenge and during the entire 3 min of hypoxic challenge and expressed as impulses per second unless otherwise stated. In anesthetized animals, the following respiratory variables were analyzed: RR (phrenic bursts per minute), amplitude of the integrated tidal phrenic nerve activity (a.u.), and MNR [number of phrenic bursts/min (RR) × amplitude of integrated tidal phrenic nerve activity (a.u.)]. Data are presented as individual data points along with means ± SEM, unless otherwise stated. The following statistical methods were used. If the data met normal distribution (Shapiro-Wilk test) and equal variances (Levene’s median test), then a *t* test or one-way analysis of variance (ANOVA) followed by a post hoc test was performed. If the data did not meet the above criterion, then Mann-Whitney rank sum test or one-way ANOVA on ranks followed by a post hoc test was performed. To determine whether the means of two or more groups are affected by two different factors (genotype and treatment/time point/dose), two-way ANOVA or two-way ANOVA with repeated measures was performed. Nonlinear regression was performed to determine the EC_50_ of the effect of NaHS on cAMP levels in HEK293 cells. All statistical analyses were performed using Sigma Plot (version 11), and *P* values of <0.05 were considered significant.

## Notes

View/request a protocol for this paper from *Bio-protocol*.
