# Supplementary material for: Hypoxia sensing requires H2S-dependent persulfidation of olfactory receptor 78
Source: Sci Adv. 2023 Jul 5;9(27):eadf3026. doi: 10.1126/sciadv.adf3026 (PMC10321732; doi:10.1126/sciadv.adf3026)
Supplement: Supplementary file 1 — Figs. S1 to S19 Tables S1 to S4 [file sciadv.adf3026_sm.pdf]

Science Advances
AAAS

Supplementary Materials for

Hypoxia sensing requires H_2S-dependent persulfidation of olfactory receptor 78

Ying-Jie Peng *et al.*

Corresponding author: Nanduri R. Prabhakar, nanduri@uchicago.edu

Sci. Adv. **9**, eadf3026 (2023)
DOI: 10.1126/sciadv.adf3026

This PDF file includes:

Figs. S1 to S19
Tables S1 to S4

SUPPLEMENTARY FIGURES AND LEGENDS

fig. S 1

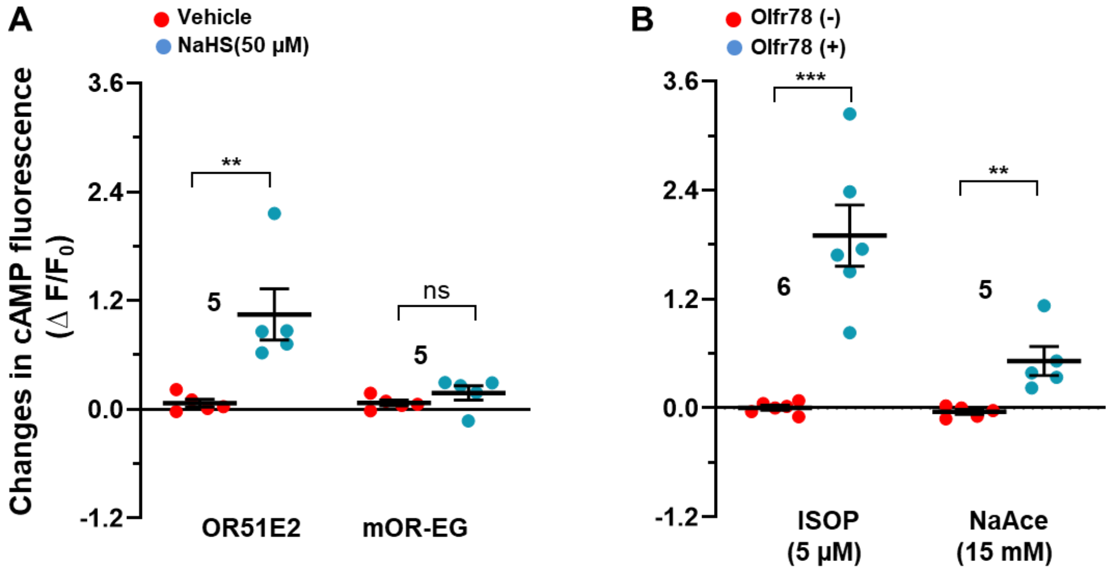

figure S1. NaHS increases cAMP fluorescence in HEK293 cells expressing OR51E2. Intensity of cAMP fluorescence was measured with a fluorescence plate reader and presented as the ratio of absolute change (stimuli-baseline) and baseline (ΔF/F0). (**A**) Cells expressing the human ortholog OR51E2 of mouse Olfr78 or the unrelated mouse odorant receptor mOR-EG were exposed to 50 μM NaHS or vehicle and the change in the intensity of cAMP fluorescence was determined during the entire 5 min after NaHS application. (**B**) cAMP response of cells transfected with Olfr78 (+) but not with vector plasmids (-) to the adrenergic receptor agonist isoproterenol (ISOP, 5μM) or the Olfr78 ligand sodium acetate (NaAce, 15mM) during the entire 5 min after application. Numbers above the bars represent the number of experiments. **, *P* < 0.01; ***, *P*<0.001; ns, *P* > 0.05; Mann-Whitney test, or *t*- test (ISOP).

fig. S 2

			Cysteine residue
msscnfthat	flligipgle	eahfwfgfpl	4
lsmyavalfg	ncivvfivrt	erslhapmyl	42
flcmlaaidl	alststmpki	lalfwfdsre	63
itfdaclaqm	ffihtlsaie	stillamafd	96
ryvaichplr	haavlnntvt	vqigmvalvr	126
gslfffplpl	likrlafchs	nvlshsycvh	168,178
qdvmklaytd	tlpnvvyglt	aillvmgvdv	
mfislsyfli	irtvlqlpsk	serakafgtc	240
vshisvvlaf	yvpliglsvv	hrfgnsldpi	
vhvlmgdvyl	llppvinpii	ygaktkqirt	
rvlamfkisc	dkdieaggnt		310

figure S2. Primary amino acid sequence of mouse Olfr78 showing Cys residues. Cys residues are highlighted yellow.

fig.S3

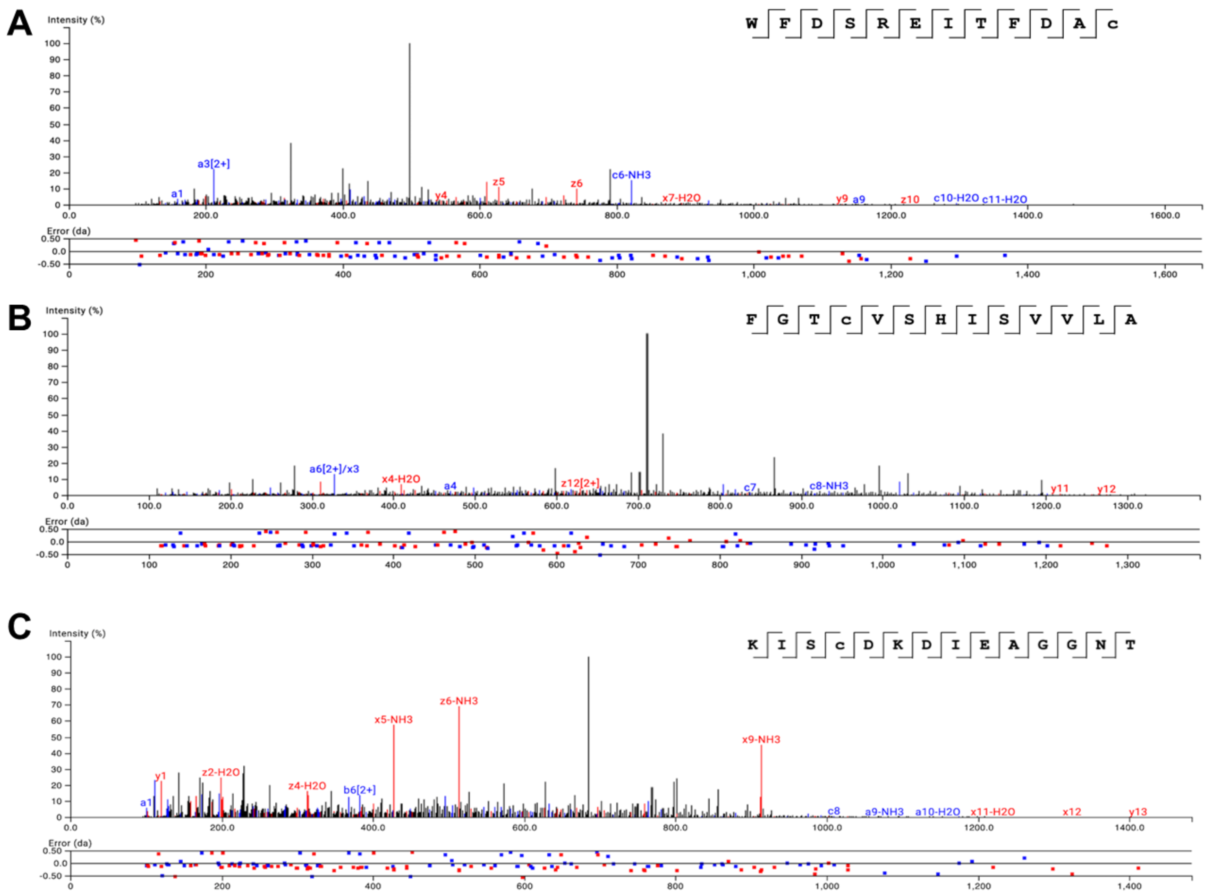

figure S3. MS/MS spectra of peptides containing persulfidated Cys residues. A) Cys^{96} peptide containing CyS-S-IAM label (m/z +89.09). **B**) Cys^{240} peptide containing CyS-S-IAM label (m/z +89.09). **C**) Cys^{310} peptide containing CyS-S-IAM label (m/z +89.09).

fig. S 4

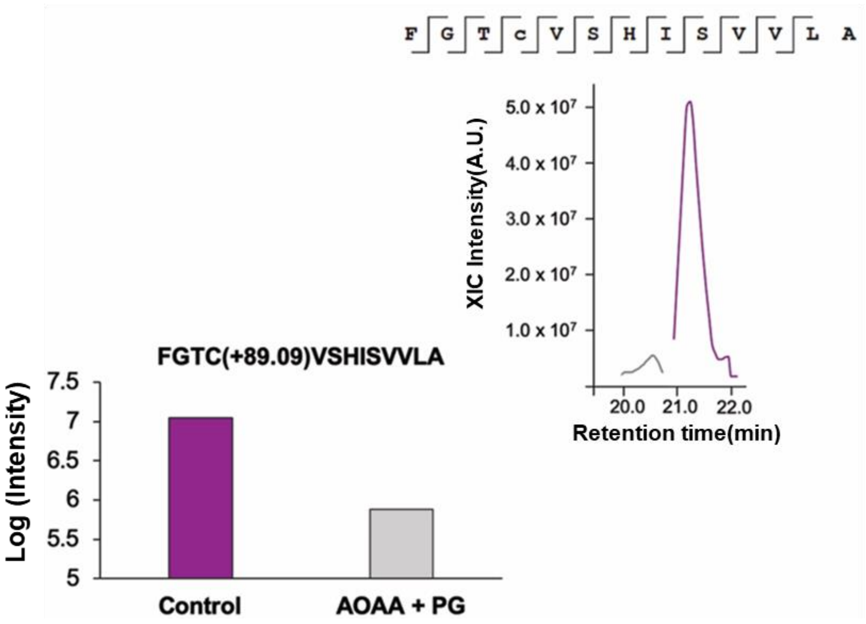

figure S4. Quantification of persulfidated Cys240 peptide of Olfr78 isolated from HEK cells treated with 1 mM aminooxyacetic acid (AOAA) and propargyl glycine (PG) or vehicle (control). Inlet representing fragmentation and extracted ion chromatogramme (XIC) is shown.

fig. S 5

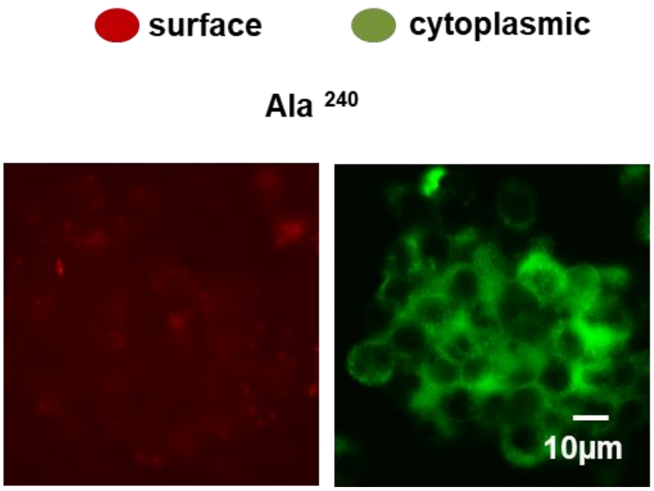

figure S5. Substituting Cys^{240} with alanine impairs trafficking of Olfr 78 to the membrane in HEK-293 cells. Cell surface detection of live cells (*surface*) and in the cytosol (*cytoplasmic*) Scale: 10µm. N=4 individual experiments.

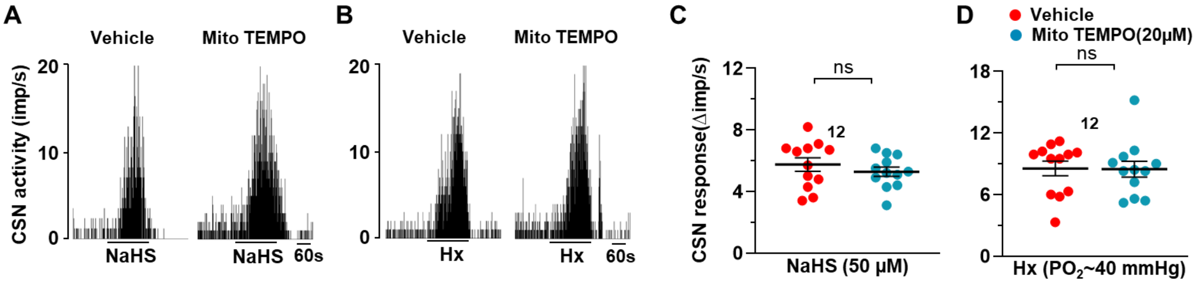

figure S6. Carotid body sensory nerve (CSN) responses to NaHS and hypoxia are unaltered by mito tempo. (**A-B**) Examples of carotid body sensory nerve (CSN) responses to 50 μM NaHS (**A**) or hypoxia (Hx; PO_2 ~40 mmHg, **B**) in presence of vehicle or Mito Tempo from a wild type mouse. (**C-D**) Average (mean ± SEM) and individual data of CSN responses to NaHS (**C**) and hypoxia (Hx ; **D**) expressed as stimulus evoked minus baseline CSN activity (Δimpulses/s). Numbers (n) represent the number of carotid bodies. ns, *P*>0.05, paired *t*-test.

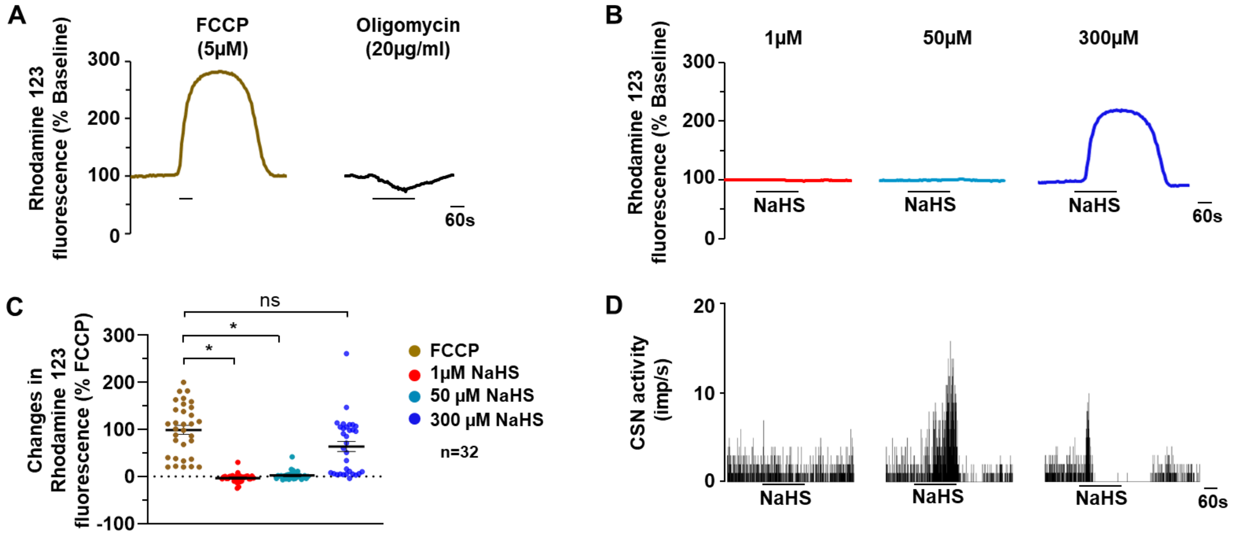

figure S7. Mitochondrial membrane potential (MMP) responses of mice glomus cells to NaHS. (**A-B**) Examples Rhodamine 123 fluorescence intensity (% baseline) in response to 5 µM FCCP (1 min, **A**), 20 µg/ml Oligomycin complex (3 min, **A**) and 1, 50, and 300 µM NaHS (each 3 min, **B**) in a glomus cell from wild type mouse. (**C**) Average (mean ± SEM) and individual data of changes in Rhodamine 123 fluorescence intensity in response to 1, 50, and 300 µM NaHS in mice glomus cells normalized to percentage of the responses evoked by FCCP. n=32 cells. *, P<0.05; ns, P >0.05; one-way ANOVA on Ranks followed by Dunn test. (**D**) Examples of CSN responses to 1, 50, and 300 µM NaHS from a wild type mouse.

fig.S8

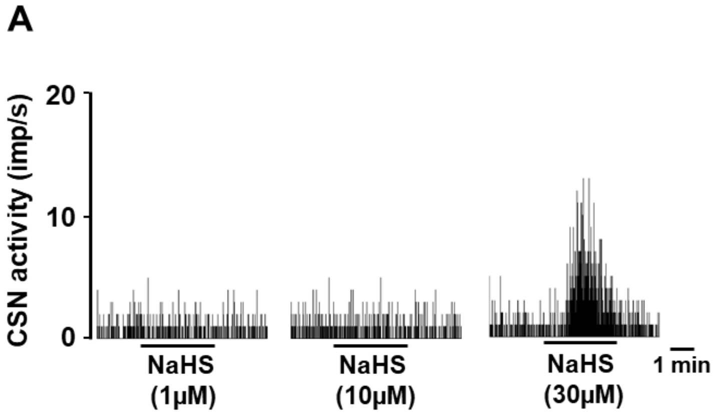

figure S8. Carotid body sensory nerve (CSN) responses to NaHS. (**A**) Examples of CSN responses to 1, 10, and 30 µM NaHS of *ex vivo* carotid bodies from wild type mouse. Black bars represent duration of NaHS application. (**B**) Average (mean ± SEM) and individual data of CSN response to NaHS are shown. Data presented as stimulus evoked minus baseline CSN activity (∆impulses/s). n=13 carotid bodies. *, $P<0.05$; ns, $P>0.05$; one-way ANOVA on Ranks followed by Dunn test.

fig. S9

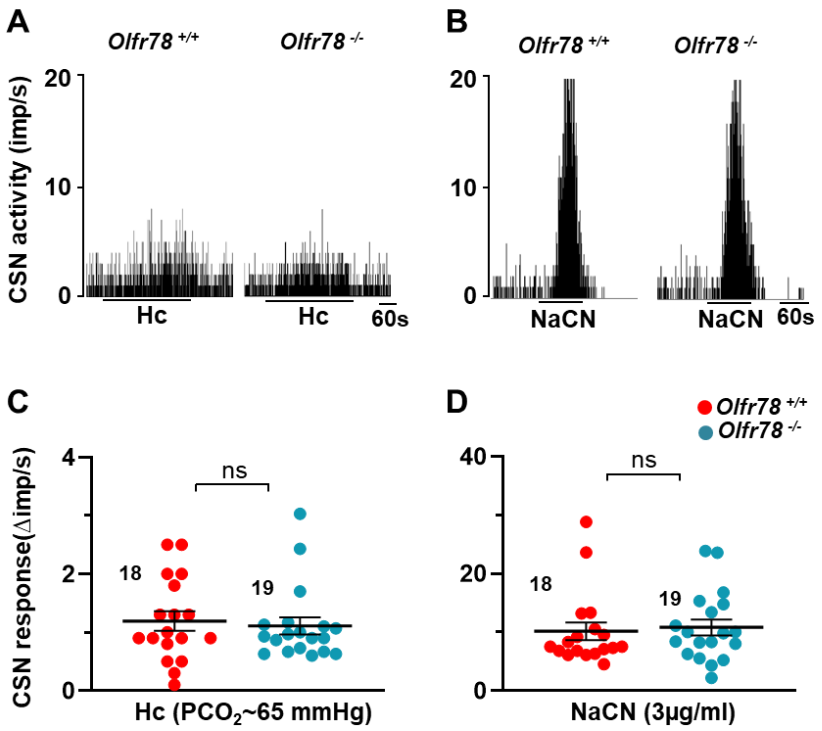

figure S9. Comparable carotid body sensory nerve (CSN) responses to CO_2 and NaCN in *Olfr78*$^{+/+}$ and *Olfr78* $^{-/-}$ mice. CSN responses to hypercapnia (Hc; **A**) and NaCN (**B**). Hypercapnic (Hc) application was for 5min (Hc, PCO_2= 65 mmHg), and to NaCN (3µg/ml) was applied for 90 s. Black bars represent duration of hypercapnia and NaCN application. (**C-D**) Average (mean ± SEM) and individual data of CSN response to hypercapnia (**C**), and to NaCN (**D**) are shown. Data presented as stimulus minus baseline CSN activity (Δimpulses/s). Numbers in C and D represent number of carotid bodies. ns, $P > 0.05$. Mann-Whitney test.

fig. S10

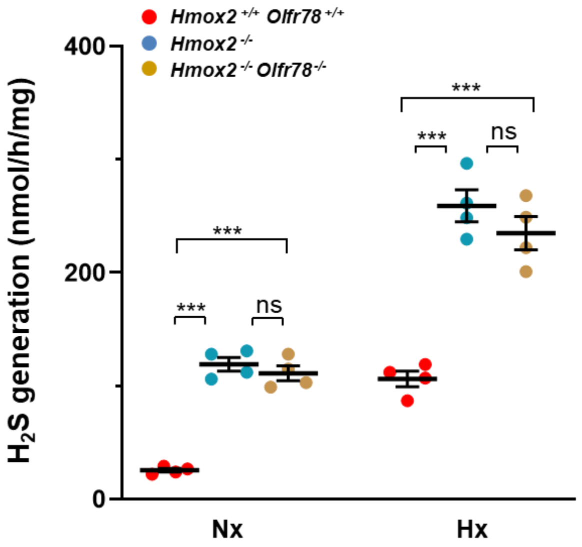

figure S10. H_2S abundance of *Hmox-2/Olfr78* double null mice carotid bodies. Average data (mean ± SEM) of H_2S generation in carotid bodies exposed to normoxia (Nx, medium P_{O_2} ~140 mmHg) and hypoxia (Hx, medium P_{O_2} ~40 mmHg) from mice of the indicated genotypes. n = 4 experiments. ***, $P < 0.001$; ns, $P > 0.05$, two-way ANOVA followed by Holm-Sidak test.

fig. S11

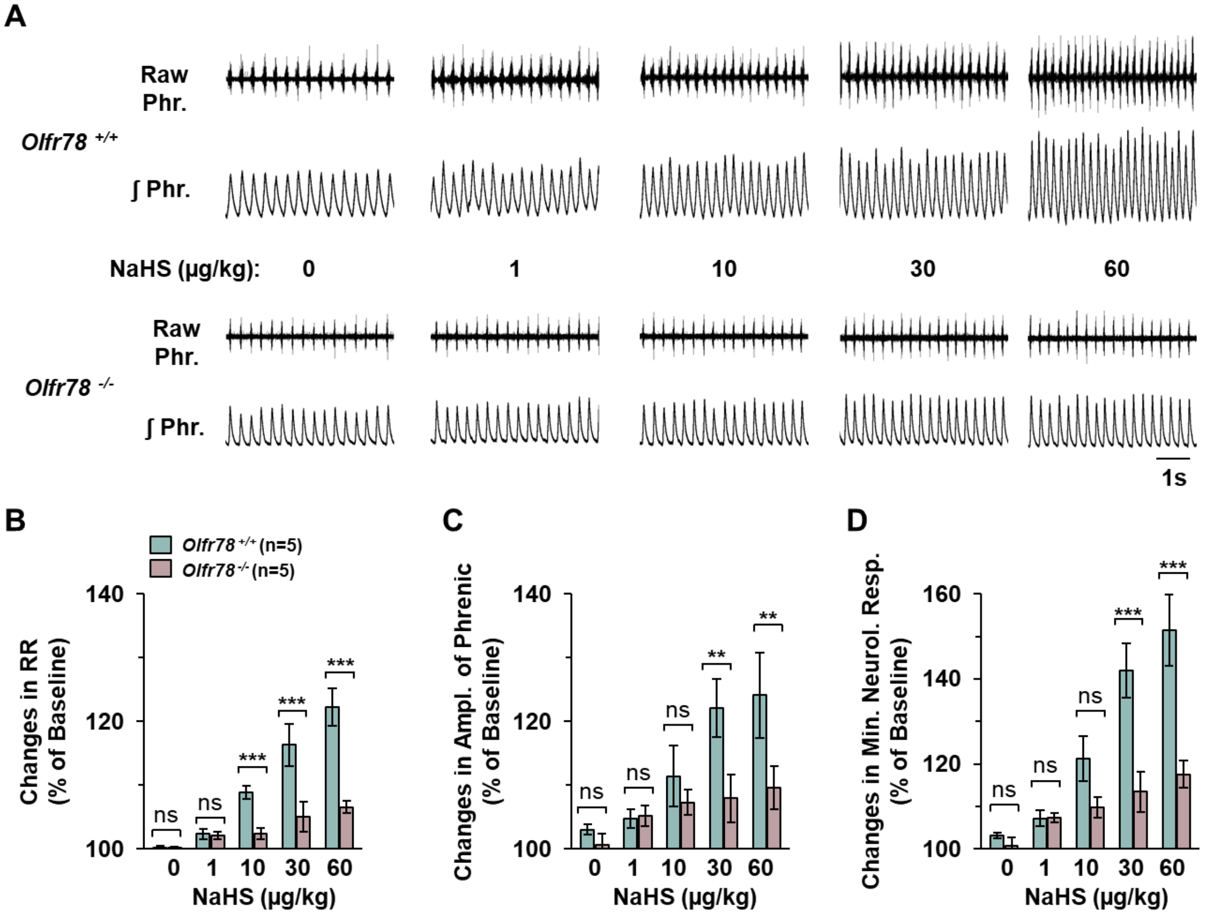

figure S11. Phrenic nerve responses to NaHS in anesthetized wild type and Olfr78 mutant mice. (**A**) Example tracings of efferent phrenic nerve activity in response to the indicated doses of NaHS in anesthetized and spontaneously breathing *Olfr78*$^{+/+}$ and *Olfr78*$^{-/-}$ mice. Body temperature of mice was kept at 38±1ºC, and each concentration of NaHS was administrated within 20 s through a catheter inserted into right external jugular vein. Raw Phr., raw phrenic nerve activity. ∫Phr., integrated phrenic nerve activity (arbitrary units). (**B-D**) Quantitative data (mean ±SEM) of phrenic nerve responses to the indicated concentrations of NaHS presented as percentage of the activity before administration of NaHS or vehicle (% of baseline). Numbers represent the number

of mice in each genotype. **, $P < 0.01$; ***, $P<0.001$; ns, $P > 0.05$, two-way ANOVA with repeated measures followed by Holm-Sidak test.

fig.S12

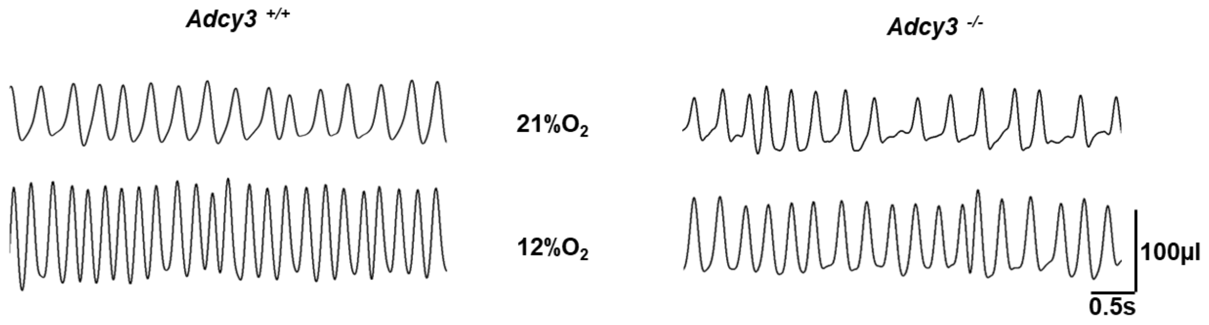

figure S12. Hypoxia ventilatory response is impaired in *Adcy3* $^{-/-}$ mice. Example tracings of breathing measured by whole body plethysmography in *Adcy3* $^{+/+}$ and *Adcy3* $^{-/-}$ mice breathing 21% and 12% O_2.

	Adcy3 $^{+/+}$ (n=7)		*Adcy3* $^{-/-}$ (n=7)	
	**21%O$_2$**	**12%O$_2$**	**21%O$_2$**	**12%O$_2$**
**RR (breaths/min)**	184±3	258±6	186±3^{ns}	217±3 ***
**V_T (µl/g)**	2.73±0.16	3.53±0.21	2.79±0.18^{ns}	3.19±0.15^{ns}
**V_E (ml/g·min)**	0.50±0.03	0.91±0.07	0.52±0.03^{ns}	0.69±0.03 *
**V_{O2} ((ml/g·min)**	0.102±0.012	0.060±0.002	0.102±0.008^{ns}	0.058±0.005^{ns}
**V_{CO2} (ml/g·min)**	0.073±0.007	0.061±0.003	0.075±0.004^{ns}	0.066±0.005^{ns}
**V_E / V_{O2}**	5.01±0.23	15.95±0.94	5.45±0.71^{ns}	11.56±0.56***

table S1. Absolute values of breathing and metabolic variables in *Adcy3* $^{+/+}$ and *Adcy3* $^{-/-}$ mice breathing 21% and 12%O$_2$. RR respiratory rate; V_T tidal volume; V_E minute ventilation; V_{O2}, oxygen consumption; V_{CO2}, CO_2 production; V_E/V_{O2}, ratio of minute ventilation / oxygen consumption. Number in parentheses represent number of mice. *, $P < 0.05$; ***, $P<0.001$; ns, $P > 0.05$, two-way ANOVA with repeated measures followed by Holm-Sidak test.

fig. S13

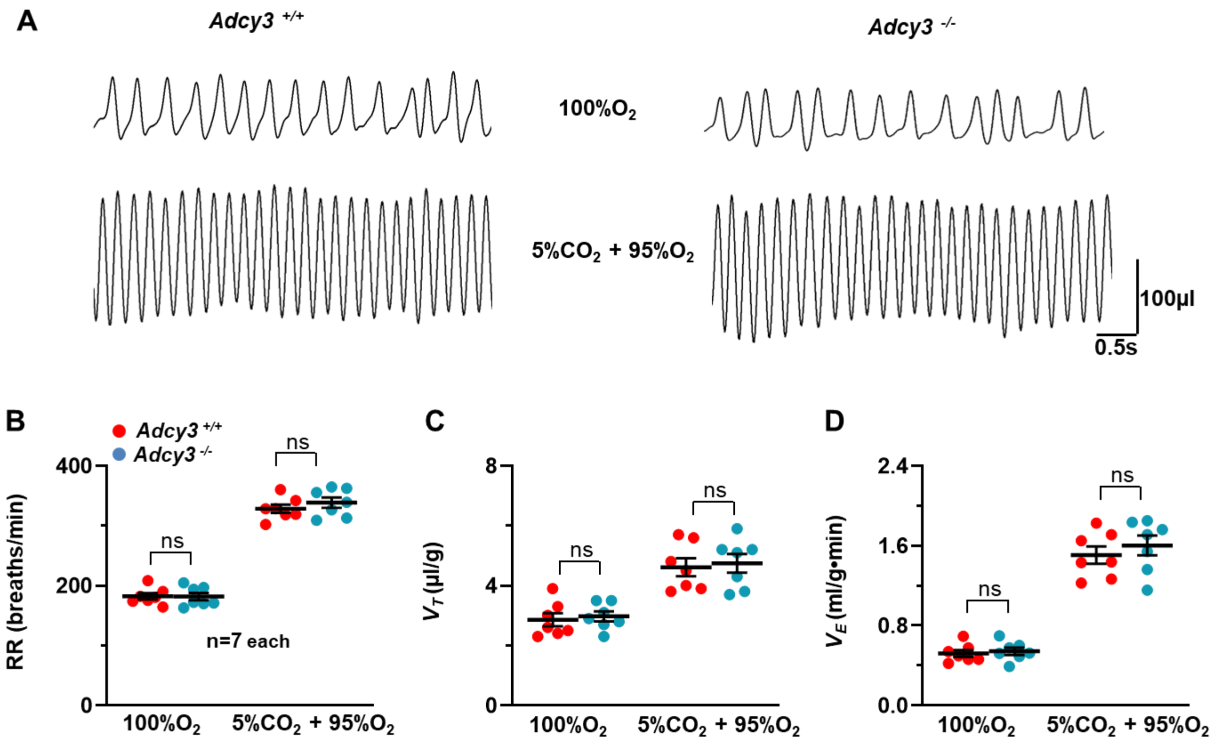

figure S13. Ventilatory response to 5% CO_2 in *Adcy3* mutant. (**A**) Example tracings of breathing measured by whole body plethysmography in Adcy*3* $^{+/+}$ and *Adcy3* $^{-/-}$ mice breathing 100% O_2 and 5% CO_2 + 95% O_2. **B-D**) Average and individual data of respiratory rate (RR; breaths/min; **B**); tidal volume normalized to body weight (V_T, µl/g, **C**); minute ventilation normalized to body weight (V_E, ml/g.min, **D**). n= 7 mice for each genotype. ns, $P > 0.05$, two-way ANOVA with repeated measures followed by Holm-Sidak test.

	Adcy3 $^{+/+}$ (n=7)		*Adcy3* $^{-/-}$ (n=7)	
	**100%O_2**	**95%O_2+5%CO_2**	**100%O_2**	**95%O_2+5%CO_2**
**RR (breaths/min)**	182±5	328±7	182±6^{ns}	339±9^{ns}
**V_T (µl/g)**	2.88±0.22	4.6±0.29	2.99±0.17^{ns}	4.75±0.32^{ns}
**V_E (ml/g·min)**	0.52±0.03	1.51±0.09	0.54±0.04^{ns}	1.60±0.10^{ns}

table S2. Absolute values of breathing responses of *Adcy3* $^{+/+}$ and *Adcy3* $^{-/-}$ mice breathing 100% O_2 and 5% CO_2. RR respiratory rate; V_T tidal volume; V_E minute ventilation. Number in parentheses represents number of mice. ns, $P > 0.05$, two-way ANOVA with repeated measures followed by Holm-Sidak test.

fig. S14

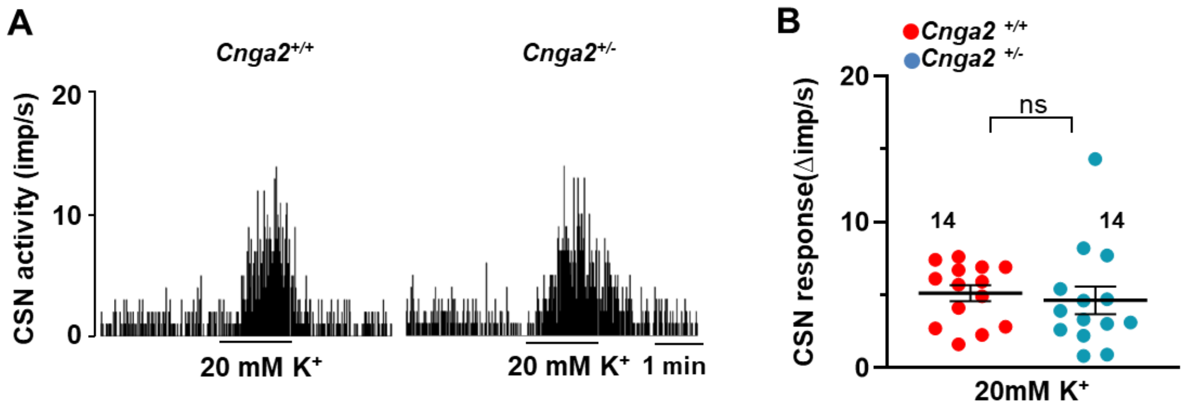

figure S14. High K^+ equally stimulates carotid body sensory nerve (CSN) activity in *Cnga2*$^{+/+}$ and *Cnga2*$^{+/-}$ mice. (**A**) Examples of CSN responses to 20mM KCl. Black bars represent duration of K^+ application. (**B**) Average (mean ± SEM) and individual data of CSN responses to 20 mM K^+ expressed as K^+-evoked CSN activity minus baseline activity (Δimpulses/s). Numbers in the represent the number of carotid bodies. *Cnga2*$^{+/+}$ *vs Cnga2*$^{+/-}$ ns, $P > 0.05$, Mann-Whitney test.

.

fig.S15

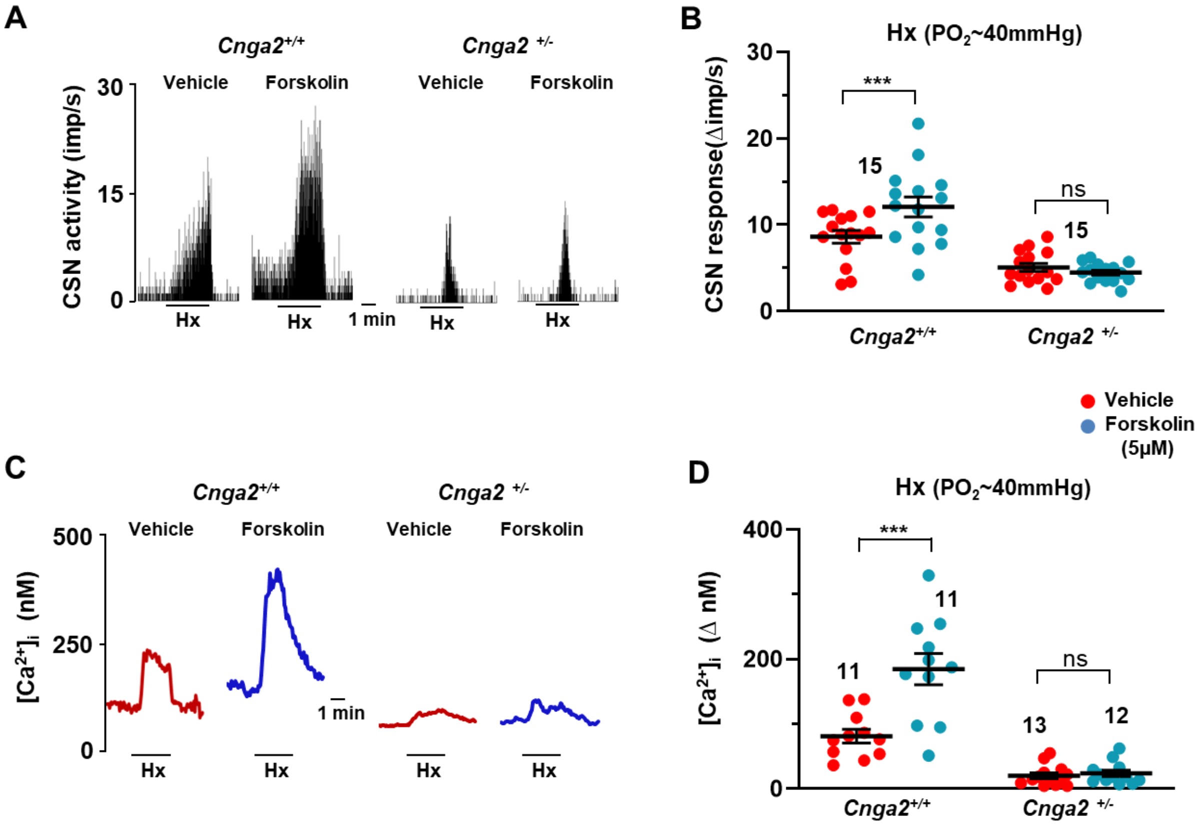

figure S15. Forskolin enhances carotid body sensory nerve (CSN) and glomus cell $[Ca^{2+}]_i$ responses to hypoxia. (**A**) Examples of CSN responses to hypoxia (Hx, P_{O_2} ~40 mmHg) in the presence of vehicle or foskolin (5µM) in *Cnga2*$^{+/+}$ and *Cnga2* $^{+/-}$ mice. (**B**) Average (mean ± SEM) and individual data of CSN responses to hypoxia expressed as hypoxia evoked response minus baseline CSN activity (Δimpulses/s). **C**) Example of $[Ca^{2+}]_i$ response to hypoxia (Hx) of glomus cells from *Cnga2* $^{+/+}$ and *Cnga2* $^{+/-}$ mice and individual and average data are shown in **D**. Numbers in **B** represent number of mice and in **D** indicate number of cells. ***, $P < 0.001$and ns, $P>0.05$ two-way ANOVA followed by Holm-Sidak test (**D**), or two-way ANOVA with repeated measures followed by Holm-Sidak test (**B**).

fig. S16

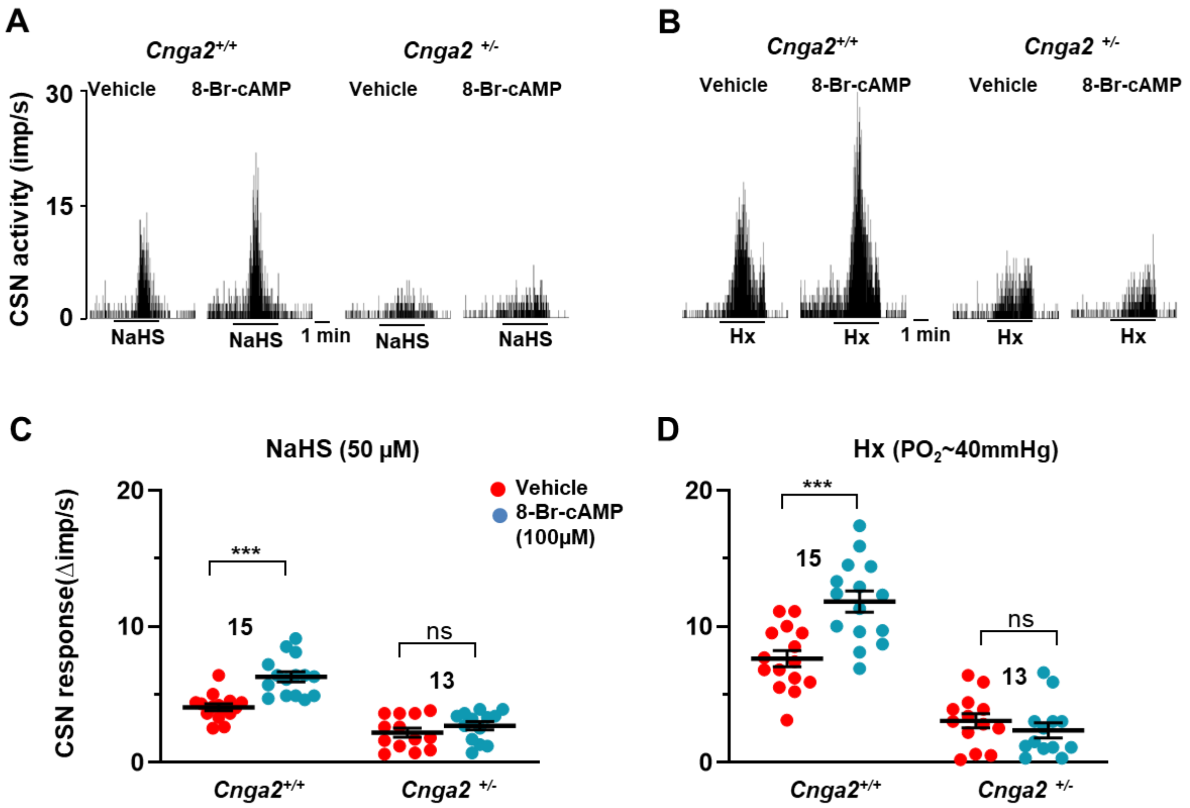

figure S16. 8-bromo cAMP enhances carotid body sensory nerve (CSN) responses to NaHS and hypoxia. (**A-B**) Examples of CSN responses to NaHS (50µM) (**A**) and hypoxia (Hx, pO_2 ~40 mmHg) (**B**) in the presence of vehicle or 8-bromo cAMP (100µM) in *Cnga2*$^{+/+}$ and *Cnga2* $^{+/-}$ mice. (**C-D**) Average (mean ± SEM) and individual data of CSN responses to NaHS (**C**) and hypoxia (Hx, PO_2 ~40 mmHg in **D**) expressed as stimulus evoked response minus baseline CSN activity (Δimpulses/s). Numbers (n) represent the number of carotid bodies. ***, $P < 0.001$ and ns, $P>0.05$ two-way ANOVA with repeated measures followed by Holm-Sidak test.

fig.S17

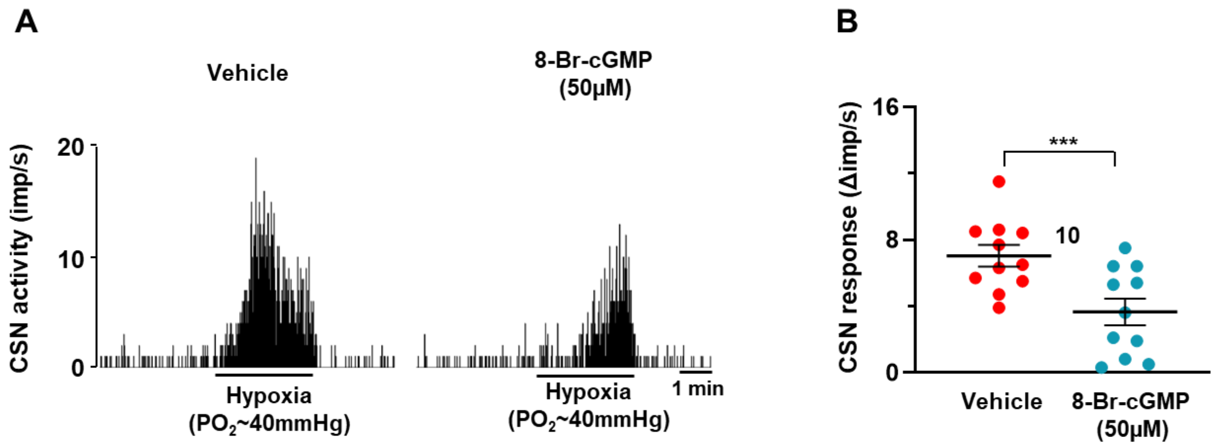

figure S17. 8-Br-cGMP attenuates carotid body sensory nerve (CSN) hypoxia. (**A**) Examples of CSN responses to hypoxia (Hx, P_{O_2} ~40 mmHg) in presence of vehicle or 8-Br-cGMP (50µM) in a wild type mouse. (**B**) Average (mean ± SEM) and individual data of CSN responses to hypoxia expressed as stimulus evoked minus baseline CSN activity (Δimpulses/s). Numbers in **B** represent the number of carotid bodies. *******, *P* < 0.001; paired *t*-test.

fig.S18

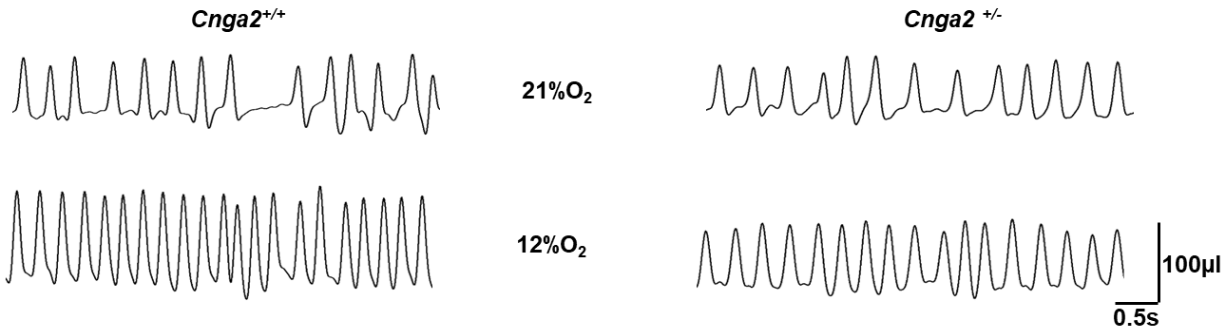

figure S18. Hypoxic ventilatory response is impaired in *Cgna2* $^{+/-}$ mice. Example tracings of breathing measured by whole body plethysmography in unanesthetized *Cgna2* $^{+/+}$ and *Cgna2* $^{+/-}$ mice breathing 21% and 12% O_2.

	Cnga2 $^{+/+}$ (n=7)		*Cnga2* $^{+/-}$ (n=7)	
	21%O_2	12%O_2	21%O_2	12%O_2
RR (breaths/min)	163±5	235±4	166±2 ^{ns}	209±4 ***
V_T (µl/g)	2.91±0.08	3.52±0.09	2.87±0.09^{ns}	3.14±0.10 *
V_E (ml/g·min)	0.48±0.02	0.83±0.03	0.48±0.02^{ns}	0.66±0.03 ***
V_{O2} ((ml/g·min)	0.084±0.004	0.048±0.004	0.079±0.003^{ns}	0.052±0.002^{ns}
V_{CO2} (ml/g·min)	0.061±0.004	0.052±0.003	0.058±0.005^{ns}	0.055±0.002^{ns}
V_E / V_{O2}	5.77±0.45	18.19±1.78	6.11±0.43^{ns}	12.65±0.61 **

table S3. Absolute values of breathing and metabolic variable responses of *Cgna2* $^{+/+}$ and *Cgna2* $^{+/-}$ mice breathing 21% and 12%O_2. RR respiratory rate; V_T tidal volume; V_E minute ventilation; V_{O2}, oxygen consumption; V_{CO2}, CO_2 production; V_E/ V_{O2}, ratio of minute ventilation / oxygen consumption.. Number in parentheses represents number of mice. *, $P < 0.05$; **, $P<0.01$; ***, $P<0.001$and ns, $P > 0.05$, two-way ANOVA with repeated measures followed by Holm-Sidak test.

fig.S19

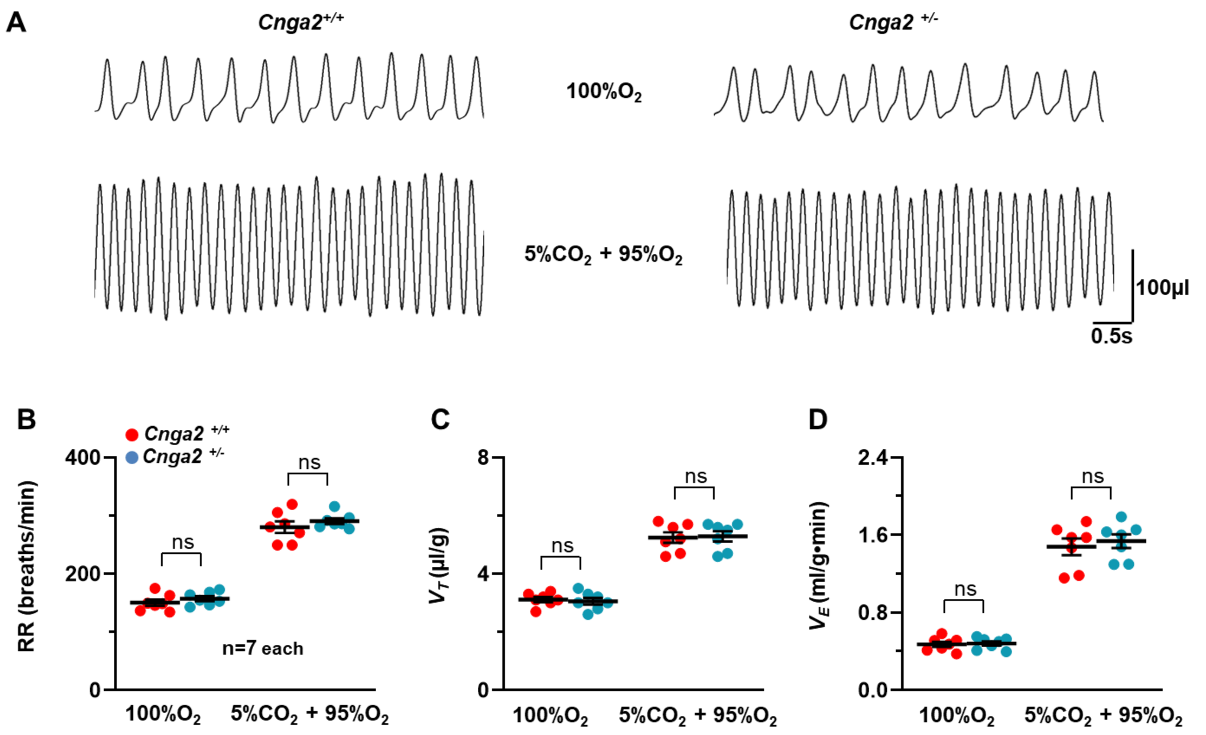

figure S19. Comparable breathing response to 5% CO_2 in *Cnga2*$^{+/+}$ and *Cnga2*$^{+/-}$ mice. (**A**) Example tracings of breathing measured by whole body plethysmography in *Cnga2* +/+ and *Cnga2* $^{+/-}$ mice breathing 100% O_2 and 5% CO_2 + 95% O_2. **B-D**) Average and individual data of respiratory rate (RR; breaths/min; **B**); tidal volume normalized to body weight ($V_{T,}$ µl/g, **C**); minute ventilation normalized to body weight ($V_{E,}$ ml/g.min, **D**). n= 7 mice for each genotype. ns, $P > 0.05$, two-way ANOVA with repeated measures followed by Holm-Sidak test.

	Cnga2 $^{+/+}$ (n=7)		*Cnga2* $^{+/-}$ (n=7)	
	**100%O_2**	**95%O_2+5%CO_2**	**100%O_2**	**95%O_2+5%CO_2**
**RR (breaths/min)**	150±5	280±10	157±4 ^{ns}	291±5 ^{ns}
**V_T (µl/g)**	3.13±0.09	5.26±0.18	3.05±0.11 ^{ns}	5.27±0.17 ^{ns}
**V_E (ml/g·min)**	0.47±0.03	1.48±0.09	0.48±0.02 ^{ns}	1.54±0.07 ^{ns}

table S4. Absolute values of ventilatory responses of *Cnga2* $^{+/+}$ and *Cnga2* $^{+/-}$ mice breathing 100% O_2 and 5% CO_2. RR, respiratory rate; V_T, tidal volume; V_E, minute ventilation. Number in parentheses represents number of mice. ns, *P* > 0.05, two-way ANOVA with repeated measures followed by Holm-Sidak test.
